# Recent Advances in Manganese(III)-Assisted Radical Cyclization for the Synthesis of Natural Products: A Comprehensive Review

**DOI:** 10.3390/molecules29102264

**Published:** 2024-05-11

**Authors:** Emre Biçer, Mehmet Yılmaz

**Affiliations:** 1 Faculty of Engineering and Natural Sciences, Sivas University of Science and Technology, 58010 Sivas, Türkiye; 2 Department of Chemistry, Faculty of Art and Sciences, Kocaeli University, 41380 Umuttepe, Türkiye; mehmet.yilmaz@kocaeli.edu.tr

**Keywords:** manganese(III), oxidative radical cyclization, natural products, intermolecular cyclization, intramolecular cyclization, tandem cyclization

## Abstract

Natural products play an important part in synthetic chemistry since they have many pharmacological properties and are used as active drug compounds in pharmaceutical chemistry. However, synthesis of these complex molecules is difficult due to the requirement of various synthetic steps, which include highly regio- and stereoselectivity. Therefore, oxidative radical cyclization assisted by manganese(III) acetate serves as an important step in obtaining spiro-, tricyclic, tetracyclic, and polycyclic derivatives of these compounds. Manganese(III)-based reactions offer a single-step regio- and stereoselective cyclizations and α-acetoxidations, reducing the number of synthetic steps. Also, the manganese(III)-mediated oxidative free radical cyclization method has been successfully applied for the synthesis of cyclic structures found in many natural products. This article presents a broad overview of manganese(III)-based radical reactions of natural products as a key step in overall synthesis. The authors have classified natural product synthesis processes assisted by manganese(III) acetate as intermolecular, intramolecular, oxidation, acetoxidation, aromatization, and polymerization reactions, respectively.

## 1. Introduction

Radical cyclization chemistry has attracted the attention of chemists in the last four decades. Manganese(III)-mediated chemistry, with different synthetic applications, is useful for the synthesis of several kinds of molecules. The metal acts as an oxidant and transfers one electron from carbon to the metal system. Thus, this oxidant is composed of carbon radicals with enolizable organic compounds, and as a result of its addition to unsaturated systems, new C–C bonds are achieved [1,2,3,4,5,6,7,8,9,10,11,12]. The first known radical reaction of manganese(III) acetate was described by Heiba, Dessau, Bush and Finkbeiner, who reported the synthesis of γ-lactones by reacting acetic acid with alkenes in the presence of manganese(III) acetate [13,14]. Similarly, aldehydes, ketones, monocarboxylic acids and their anhydrides, β-dicarbonyl compounds (such as β-diketones), β-keto esters, β-keto carboxylic acids and amides, malonic acid and its derivatives, and β-nitro carbonyl compounds are oxidized by manganese(III) acetate to attain a variety of products [15]. Manganese(III) mediation in reactions not only provides oxidative cyclization [16,17,18,19,20,21,22,23,24,25,26,27,28,29], but also enables highly diversified acetoxidation [30,31,32,33,34], halogenation [35,36], aromatization [37,38,39], oxidation [40,41], phosphonation [42,43], epoxidation [44], and also polymerization [45]. Cyclization occurs via two different mechanisms: intermolecular and intramolecular. It is worth noting that the superiority offered when using manganese(III) acetate is the visibility of the free radical reaction, since the initial color changes when the reaction is completed. Mostly acetic acid is used in these reactions due to the ease of dissolving manganese(III) acetate at lower temperatures. However, acetonitrile, ethanol, methanol, dioxane and DMSO are also used. In some reactions, copper(II) acetate is also used along with manganese(III), due to the fact that the oxidizing effect of copper(II) on primary and secondary radicals is stronger than that of manganese. The formed Cu(I) is then oxidized to Cu(II) by Mn(III), meaning that using a catalytic amount of Cu(II) is sufficient [10].

**Scheme 1:**
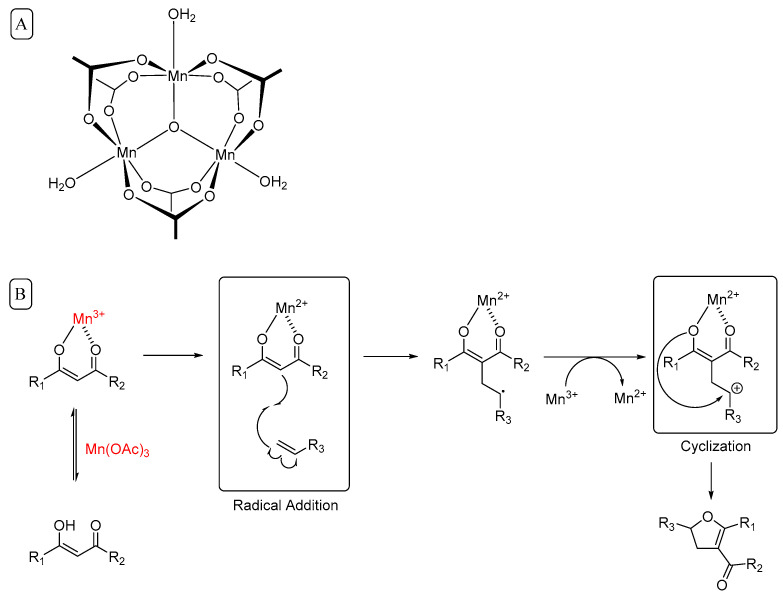
(**A**) Crystal structure of manganese(III) acetate and (**B**) single electron transfer reaction mechanism with manganese(III) acetate.

The anhydrous form of manganese(III) acetate is a linear coordination polymer with acetic acid molecules bridging between manganese atoms (Scheme 1A) [46,47]. It is usually used as a dihydrate, though the anhydrous form is also used in some situations and is slightly more reactive than the dihydrate. Manganese(III) acetate is easily prepared by reacting potassium permanganate and manganese(II) acetate in acetic acid; the addition of acetic anhydride to the reaction produces the anhydrous form [13]. Moreover, Pekel et al. disclosed a continuous electrochemical process for obtaining manganese(III) acetate with high purity [48,49].

The mechanism of reaction within manganese(III) acetate has been explained in many articles. To elucidate, the reaction begins with the formation of a manganese(III)–enolate complex followed by the reduction of Mn(III) to Mn(II), whilst a carbon radical is formed, which is attacked by an unsaturated species to form a radical intermediate. Additional manganese(III) transforms this radical to carbocation in the termination step, followed by the formation of a cyclic structure [16,17,18,19,20,21,22,23,24,25,26,27,28,29]. Oxidation occurs twice in these reactions, such that two equivalents of manganese(III) are needed to undergo these free radical cyclization reactions (Scheme 1B). A general review on free radical reactions with manganese(III) acetate was published by Nishino [6], Demir [7], Snider [8], Mondal [9], and Burton [12]. Yet, here, we wish to present manganese(III) acetate-initiated free radical reactions that target natural product synthesis.

Natural product synthesis is a developing area in synthetic organic chemistry due to its integration with medicinal and combinatorial chemistry, as well as traditional organic chemistry disciplines. Free radical chemistry constitutes the key to natural product synthesis in most forms of natural product synthesis. Particularly, manganese(III) acetate is mostly used as a radical initiator in natural product synthesis due to its versatility, ease of handling, and ready availability, alongside its regioselectivity and stereoselectivity. The purpose of this manuscript is to provide an overview of the pivotal role played by the manganese(III) acetate compound in the synthesis of natural products, targeting researchers and academics engaged in the area of study. To our knowledge, there has been no prior publication addressing this specific aspect. It is our anticipation that this manuscript will serve as a valuable and comprehensive resource, offering insights and guidance to researchers actively involved in the synthesis of natural products.

## 2. Intermolecular Radical Cyclization

**Scheme 2:**
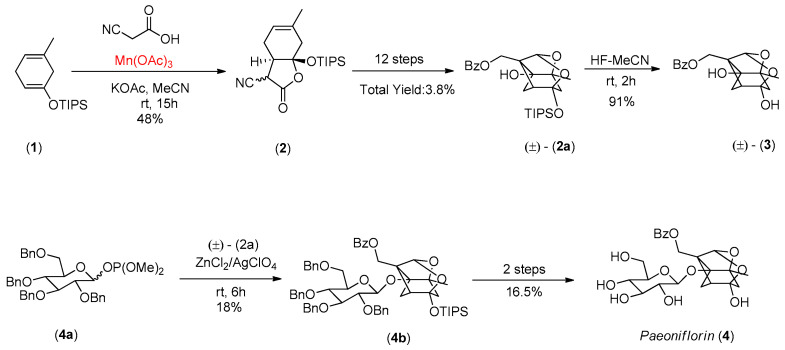
Synthesis of (±)-*Paeoniflorigenin* (**3**) and *Paeoniflorin* (**4**).

Corey et al. employed manganese(III) acetate in the initial step for the synthesis of (**2**) from dihydro-m-cresol triisopropylsilyl ether (**1**) and cyanoacetic acid, with a yield of 48%. The significance of (**2**) lies in its containing 10 carbons, crucial for the terpenoid structures of (**3**) and (**4**). A 12-step synthesis was conducted to obtain (**2a**), a precursor of (**3**), with a total yield of 3.8%. Upon the deprotection of (±)-(**2a**), the target terpenoid (±)-(**3**), (±)-*paeoniflorigenin*, was obtained. To synthesize the other natural terpenoid compound, *paeoniflorin* (**4**), β-*glycosylation* of the tertiary alcohol group of (**4a**) with the previously obtained (±)-(**2a**) yielded (**4b**). Finally, after two additional steps, *paeoniflorin* (**4**) was synthesized with a total yield of 16.5% (Scheme 2) [50].

**Scheme 3:**
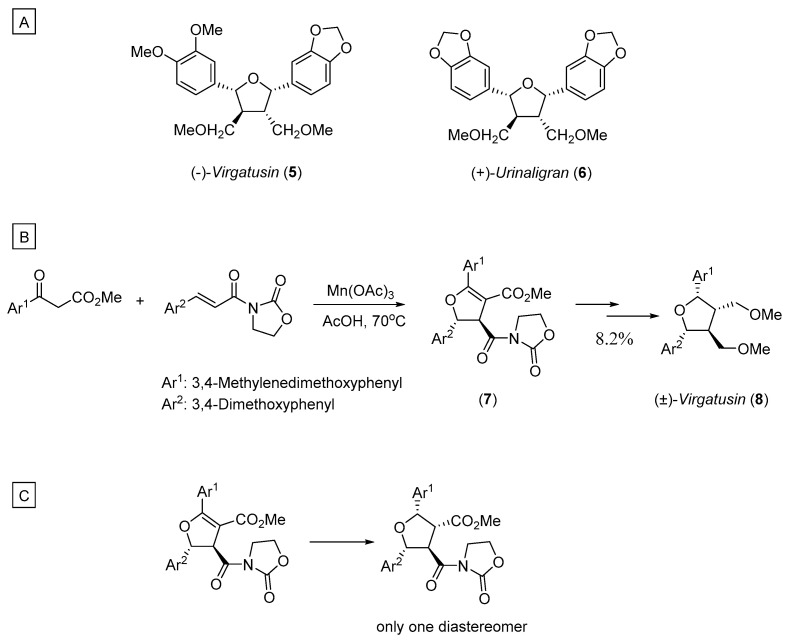
(**A**) (−)-*virgatusin* and (+)-*urinaligran*. (**B**) 2,5-Diaryltetrahydrofuran lignan derivatives and the first step of synthesis; and (**C**) hydrogenation of diastereomer.

Martinet and co-workers devised a synthetic approach to produce (−)-*virgatusin* (**5**) and (+)-*urinaligran* (**6**), both belonging to the 2,5-diaryllignan tetrahydrofuran family (Scheme 3A). Employing retrosynthetic analysis, it was determined that the synthesis could commence using appropriate cinnamate and aryl-substituted β-ketoester derivatives. In the first step, methyl benzoylacetate and N-cinnamoyloxazolidinone were employed to yield dihydrofuran (**7**). Subsequently, after three steps, a racemic (±)-*virgatusin* (**8**) was obtained with an overall yield of 20% (Scheme 3B). Additionally, it is noteworthy that in the synthesis of (+)-*urinaligran* from (*S*)-4-tert-butyl-1,3-oxazolidin-2-one, a total yield of 8.2% was achieved. Furthermore, to obtain the enantiomers of (−)-*virgatusin* and (+)-*urinaligran*, an additional five steps were required for stereochemical control. All the diastereomers exhibited a *trans*, *trans*, *cis* stereochemistry via simple Pd/C-catalyzed hydrogenation (Scheme 3C) [51].

**Scheme 4:**
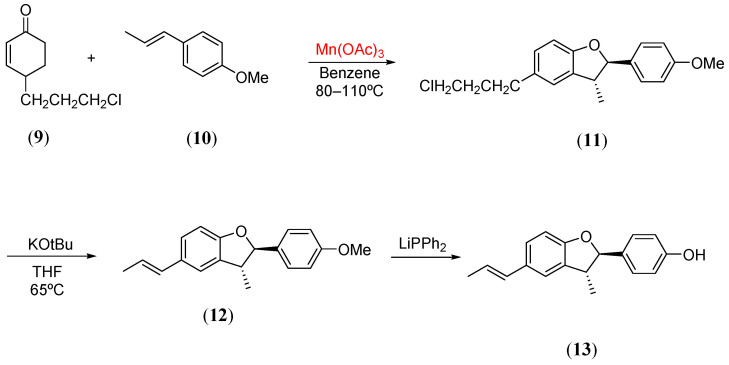
The synthesis of (±)-*conocarpan* (**13**).

Snider et al. reported the synthesis of (±)-*conocarpan* (**13**), a benzofuranoid neolignan. Intermolecular cyclization was employed to obtain an analogue structure of (±)-*conocarpan* using 2-cyclohexenone (**9**) and a styrene derivative (**10**), as illustrated in Scheme 4. Although starting with 4-allyl-2-cyclohexenone could be considered for the synthesis of (±)-*conocarpan*, complex products were obtained. Hence, 4-(3-chloropropyl)-2-cyclohexenone (**9**) was chosen to increase the yield. The overall synthesis was achieved with a yield of 19% in 4 steps [52].

**Scheme 5:**
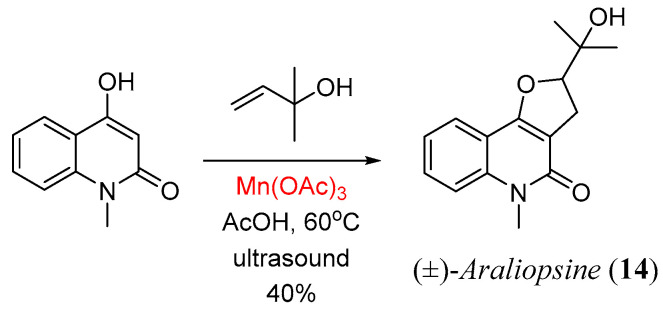
The synthesis of quinolone mimics that of (±)-*araliopsine* alkaloid (**14**).

Parsons and co-workers accomplished the synthesis of (±)-*araliopsine* (**14**) alkaloid and its analogues in a one-pot reaction with good yields, starting from quinolone derivatives. These derivatives could yield angular and/or linear tricyclic isomers depending on the substituents on the alkene, as illustrated in Scheme 5 [53]. Additionally, a subsequent study by Bar et al. investigated the synthesis of similar quinolone derivatives with various substituents, including alkyl, alkynyl, and aryl groups, resulting in yields ranging from poor to good [54].

**Scheme 6:**
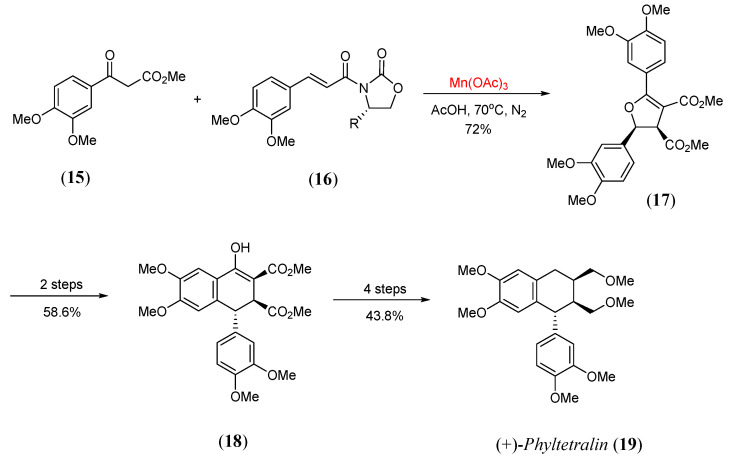
(+)-*Phyltetralin* (**19**) compound and its analogues.

Garzino has described an efficient method for the synthesis of (+)-*phyltetralin* (**19**) derivatives in four steps, with an overall yield of 19% (Scheme 6). A β-ketoester was utilized as a precursor for the synthesis, and oxidative cyclization by manganese(III) was the key step to achieve the synthesis of the target molecule [55].

**Scheme 7:**
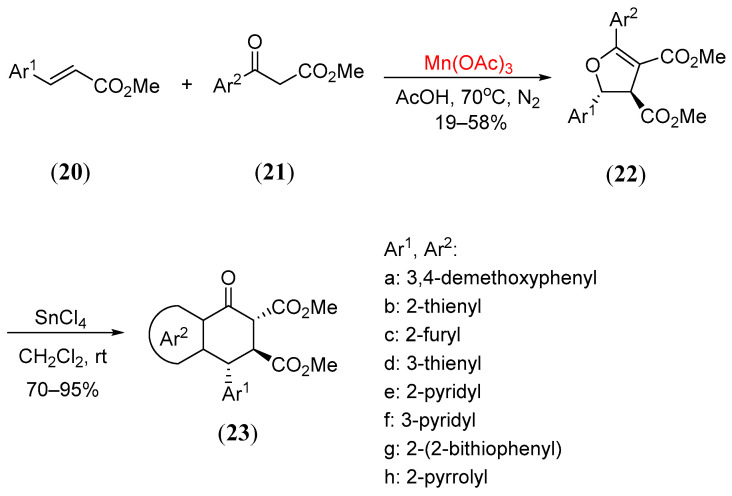
Synthesis of aryltetralin lignan compounds (**23a**–**h**).

Garzino also reported the synthesis of *heterolignans*, a pharmacologically important class of compounds. A two-step procedure was applied to obtain aryltetralin lignan derivatives (**23**) (Scheme 7). In the first step, a reaction between cinnamate derivative (**20**) and β-keto ester (**21**) yielded dihydrofuran (**22**). Subsequently, in the second step, this compound underwent rearrangement facilitated by tin(IV) chloride to afford the corresponding *heterolignans* (**23a**–**h**) [56].

**Scheme 8:**
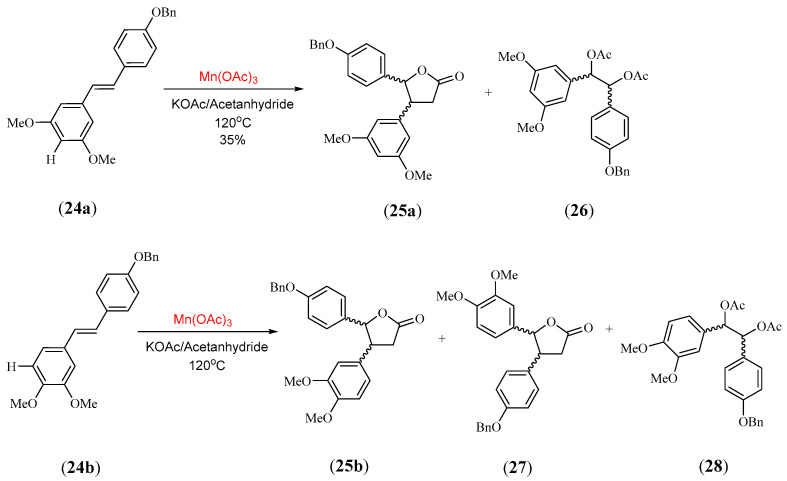
Synthesis of acetoxylated **26** and **28** and lactone **25a**, **25b** and **27**.

Thomas et al. described the synthesis of γ-butyrolactones starting from *resveratrol* analogues featuring catechol and resorcinol substitutes (Scheme 8). Following the synthesis of the corresponding lactone, ε-*viniferin*, a benzofuran derivative, could be easily obtained. The oxidative lactonization of **24a** yielded **25a** in 35% yields (as a 3:1 diastereoisomeric mixture) and **26** in 13% yield (1:1 mixture of regioisomers). Conversely, **24b** produced **25b** and **27** in a 13% yield (1:1 mixture of regioisomers) and diacetate **28** in a 12% yield [57].

**Scheme 9:**
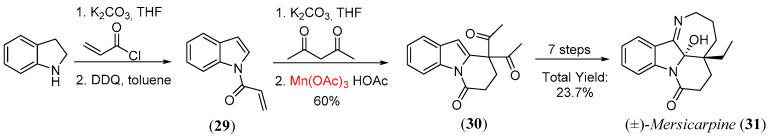
Total synthesis of (±)-*mersicarpine* (**31**) assisted by oxidative cyclization.

Magolan and Kerr successfully synthesized the indole derivative (±)-*mersicarpine* (**31**) through a synthetic procedure involving a radical addition to the double bond of N-acryl indole (**29**) to form the tricyclic skeleton (**30**) (Scheme 9). This step can be considered the key synthesis step. Subsequent reactions were conducted for the formation of other rings, ultimately leading to the successful synthesis of (±)-*mersicarpine* with a yield of 23.7% from compound (**30**). The total yield was reported as 11% from the starting compound indoline [58].

**Scheme 10:**
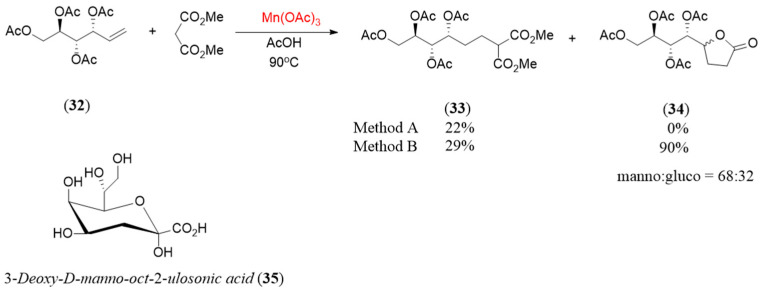
The synthesis of key intermediates of *3*-*deoxy*-*D*-*manno*-*oct*-*2*-*ulosonic acid* (**35**).

Linker and his colleagues reported the synthesis of key intermediates of *3*-*deoxy*-*D*-*manno*-*oct*-*2*-*ulosonic acid* (KDO, **35**) (Scheme 10). They utilized an acyclic alkene (**32**) derived from a carbohydrate as the starting compound, followed by a radical cyclization reaction. Excellent yields were reported. In the radical addition reaction, the addition of 10 equivalents of dimethyl malonate yielded only **33** (Method A). Conversely, without adding dimethyl malonate, a 90% yield of **34** (in a *manno:gluco* ratio of 68:32), a precursor of KDO, was obtained, along with **33** in a 29% yield (Method B). After obtaining *manno*-**34**, it could be easily transformed into KDO [59,60].

**Scheme 11:**
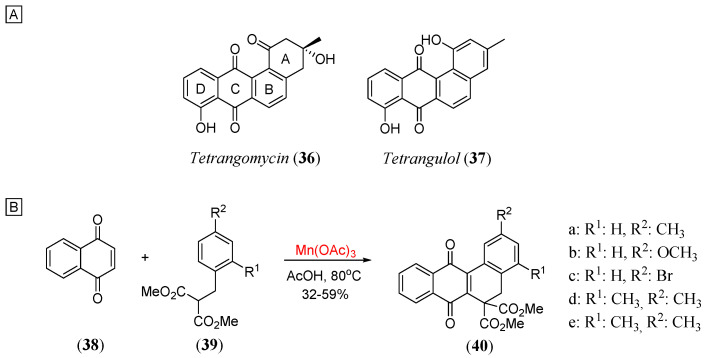
(**A**) ABCD ring of *angucyclines* (**36**) and *tetrangulol* (**37**) (**B**) Synthesis of benz[a]anthraquinone (**40**).

Carreno et al. presented a total synthesis review of *Angucyclines* involving a series of reactions, including free radical cyclization with manganese(III) acetate (Scheme 11). In the total synthesis of the target molecule, benz[a]anthraquinone (**40**) was obtained with a good yield. Consequently, the ABCD ring skeleton of *Angucyclines* was formed through the intervention of Mn(III) [61,62,63].

**Scheme 12:**
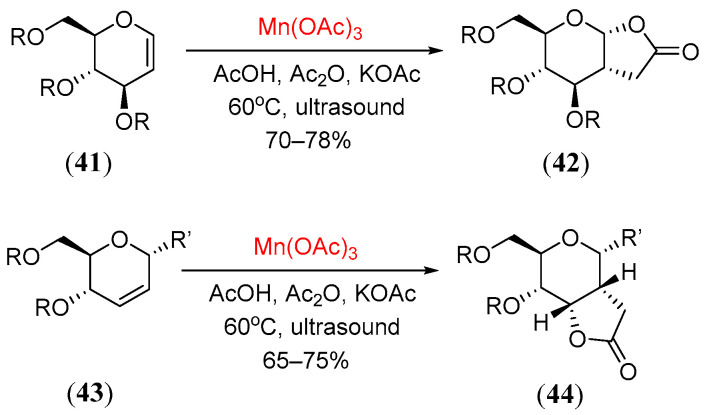
Synthesis of carbohydrate-based γ-butyrolactones.

Yousuf et al. proposed the synthesis of carbohydrate-based [4.3.0] bicyclic γ-lactone derivatives using manganese(III) acetate chemistry, starting from certain 1,2-glycal (**41**) and 2,3-glycal (**43**) derivatives (Scheme 12). The reaction exhibited moderate to good yields with no observable by-products. Additionally, a high degree of stereoselectivity was observed [64].

**Scheme 13:**
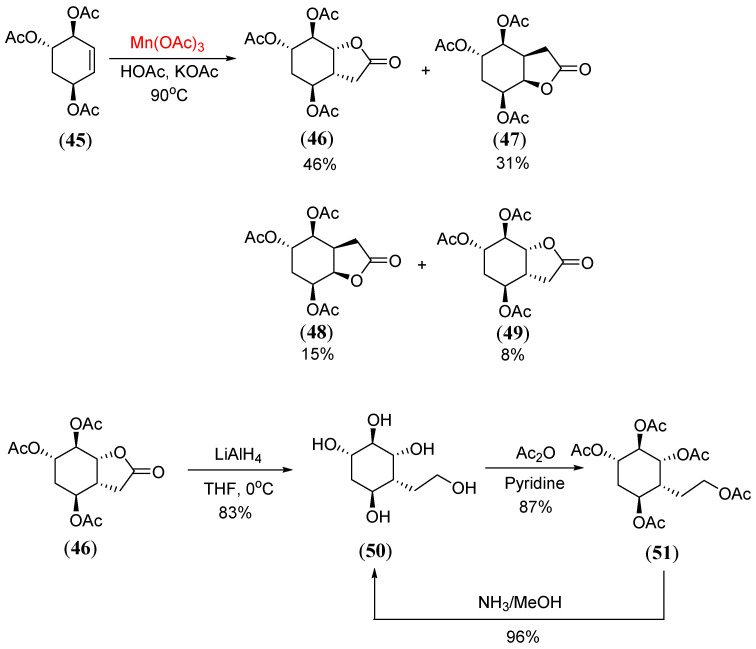
Synthesis of target carbosugar molecules.

After Yousuf’s study, Altun et al. devised a synthesis process for certain carbosugars designed to mimic carbohydrates, with potential applications in drug development. To achieve the target molecules, the key step involved an oxidative radical cyclization reaction with manganese(III) acetate, resulting in the formation of γ-lactone derivatives (**46**–**49**). Subsequent steps included the reduction of **46** and the acetylation of **50**, leading to the desired carbosugar molecule **51**. The reaction is illustrated in Scheme 13 [65].

**Scheme 14:**
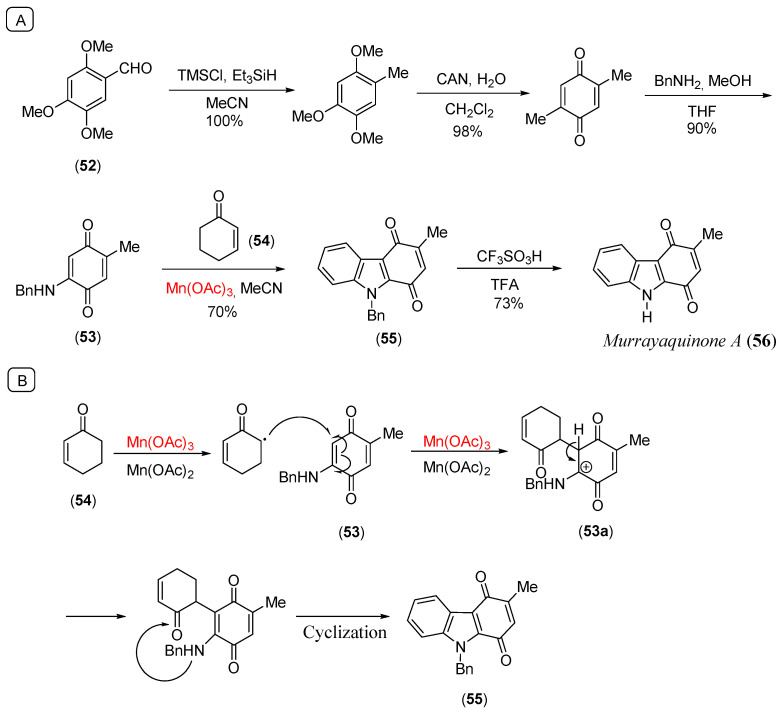
(**A**) The synthesis of *murrayaquinone A* (**56**) (**B**) Manganese(III)-based cyclization reaction of (**55**).

The other C–C bond formation reaction with the assistance of manganese(III) acetate was reported by MacDonald et al. for synthesizing *murrayaquinone A* (Scheme 14A). This reaction involved the reaction between 2-cyclohexen-1-one (**54**) and aminoquinone (**53**) via an oxidative radical reaction facilitated by manganese(III) acetate. The starting material, commercially available 2,4,5-trimethoxybenzaldehyde (**52**), underwent a three-step process to obtain the target aminobenzoquinone derivative. Subsequently, oxidative radical reaction with 2-cyclohexen-1-one yielded N-benzyl *murrayaquinone* (**55**). After an easy debenzylation with triflic acid and trifluoroacetic acid, the target natural compound *murrayaquinone A* (**56**) was obtained within five steps with an overall yield of 45%. The mechanism involves radical addition to the alkene to form the C–C bond, as illustrated in Scheme 14B [66]. Accordingly, a radical is generated with the aid of Mn(OAc)_3_. This radical then reacts with **53**, resulting in the formation of an amino-substituted carbocation **53a**. Upon further treatment with additional Mn(OAc)_3_, hydrogen abstraction occurs, leading to the formation of an enamino compound, ultimately yielding compound **55**.

## 3. Intramolecular Radical Cyclizations

### 3.1. Mono Cyclizations

**Scheme 15:**
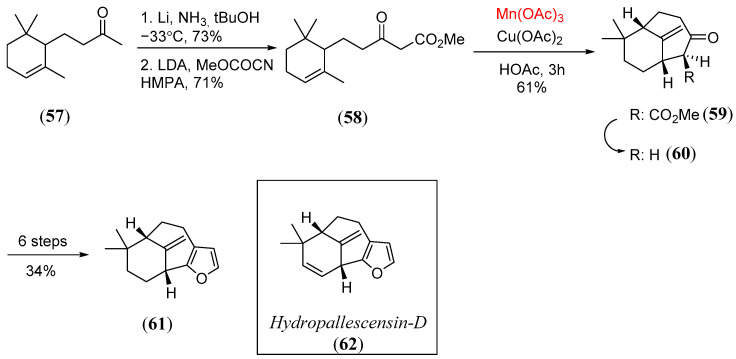
Synthesis of analog of *hydropallescensin*-*D* (**62**).

White and co-workers reported the synthesis of the furanosesquiterpene *hydropallescensin*-*D* via manganese(III) acetate-mediated cyclization (Scheme 15). They initiated the process with starting compound (**57**) to obtain the dicarbonyl compound (**58**). Subsequently, they utilized manganese(III) acetate as a radical initiator to yield the bicyclic compound (**59**), which further proceeded to form the analogue of *hydropallescensin*-*D* (**62**) in six steps with a 34% overall yield [67]. The total reaction yield was determined to be 3.2%.

**Scheme 16:**
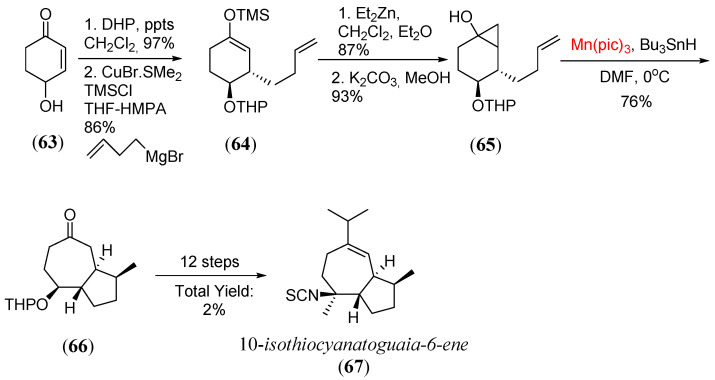
Synthesis of *10*-*isothiocyanatoguaia*-*6*-*ene* (**67**).

Iwasawa et al. disclosed the total synthesis of the isothiocyano sesquiterpene *10*-*isothiocyanatoguaia*-*6*-*ene* (**67**). A crucial step in this synthesis involved the manganese(III) free radical cyclization of bicyclo[4.1.0]heptanol (**65**) to obtain the seven-membered bicyclo[5.3.0]decane-1-one compound (**66**) (Scheme 16). This step was essential for achieving the seven-membered ring skeleton necessary for the total synthesis of *10*-*isothiocyanatoguaia*-*6*-*ene* (**67**). The reaction yielded **66** with a 76% yield and a 90% selectivity. It is noteworthy that 4-hydroxy-2-cyclohexene-1-one (**63**) was chosen as the starting molecule through retrosynthesis [68].

**Scheme 17:**
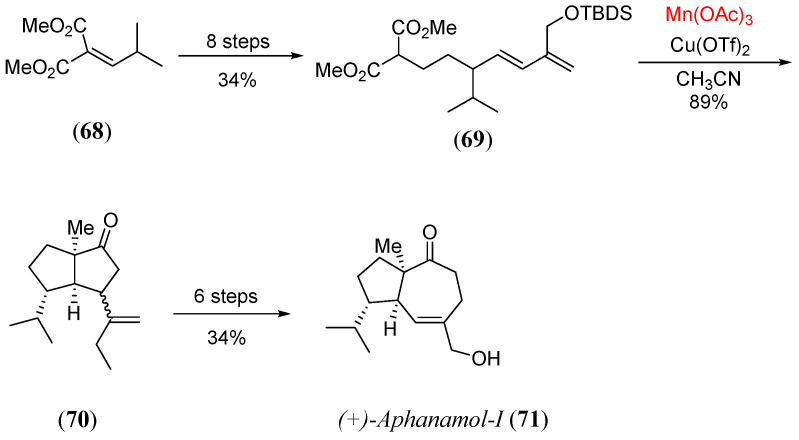
Synthesis of (+)-*aphanamol I* (**71**).

Ferrara and Burton pioneered the synthesis of the sesquiterpene (+)-*aphanamol I*, characterized by a bicyclo[5.3.0]decane core structure [69]. Burton’s efforts were primarily focused on the generation of [3.3.0]-bicyclic lactone (**70**) through oxidative radical cyclization, serving as a pivotal step in the synthesis. Beginning with an unsaturated malonate (**68**), Burton followed a sequence of six steps to procure dienyl malonate (**69**). Utilizing oxidative cyclization with manganese(III) acetate alongside copper(II) triflate in acetonitrile, [3.3.0]-bicyclic lactone (**70**) was achieved with an impressive yield of 89%. Subsequently, Burton explored two distinct approaches to the synthesis of *aphanamol I* (**71**). While the initial approach yielded a racemic mixture, the alternative method provided enantiopure (+)-*aphanamol I*. Notably, the oxidative cyclization step remained pivotal in both strategies (Scheme 17). The overall yield was determined to be 10.3%.

**Scheme 18:**
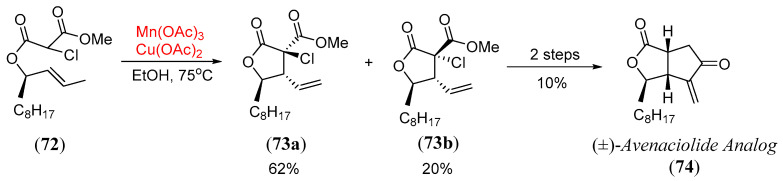
Synthesis of (±)-*avenaciolide* (**74**).

Snider et al. utilized a free radical reaction catalyzed by manganese(III) acetate to synthesize (±)-*avenaciolide* (**74**), starting from an α-chloromalonate (**72**). This compound underwent conversion into intermediate enantiomers **73a** and **73b**, yielding 62% and 20%, respectively (Scheme 18). Following this pivotal cyclization step, the final natural product (±)-*avenaciolide* analog (**74**) was efficiently obtained within a concise series of steps [70]. Subsequently, it underwent further transformation into the *avenaciolide* natural compound through methylation, as reported by Johnson [71].

**Scheme 19:**
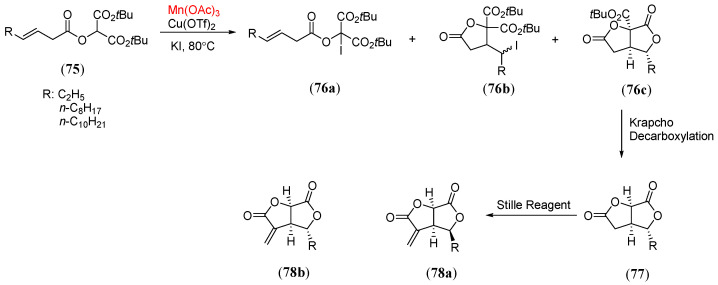
Formation of *avenaciolide* (**78**) by manganese(III)- and iodide-assisted lactonization.

Martinez and Burton also undertook the synthesis of *avenaciolide* and its derivatives [72]. Their approach involved the formation of the fused lactone (**76c**) as the primary product. To achieve this, they implemented iodine subtraction to the intermediate product to prevent the formation of other undesired products (**76a** and **76b**), resulting in the successful production of the final fused bis lactone (**76c**) with a yield of 78% (Scheme 19). Subsequently, they applied Krapcho decarboxylation followed by the Johnson protocol to attach the *exo*-methylene group, leading to the formation of *avenaciolide* (**78a** and **78b**) and its derivatives. This synthesis procedure was completed in five or six steps, with manganese(III) and iodine-assisted cyclization and lactonization serving as key steps.

**Scheme 20:**
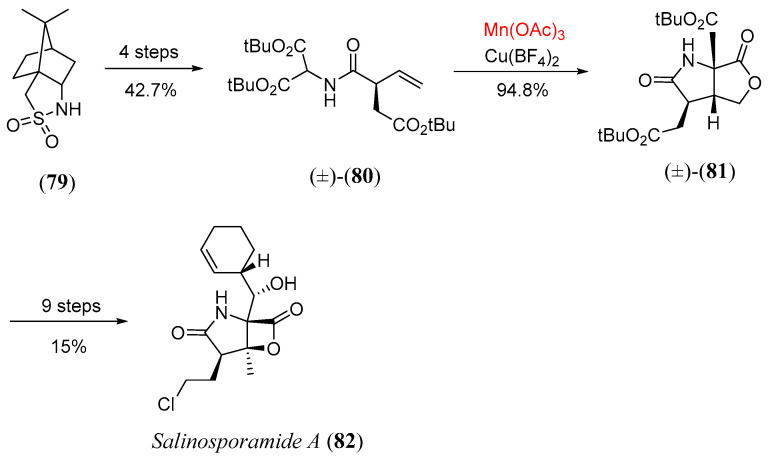
*Salinosporamide A* (**82**) synthesis with oxidative radical cyclization as a key step.

Burton et al. reported another mode of lactonization synthesis containing a [3,2,0]-bicyclic core, utilizing manganese(III) acetate in oxidative radical cyclization as a key step [73,74,75]. *Salinosporamide A*, a marine-derived natural product with potential use as a proteasome inhibitor in cancer research, served as the target molecule. Burton initiated the synthesis of *salinosporamide A* using a commercially available sultam (**79**). In four steps, amide (±)-**80** was obtained, followed by oxidative radical cyclization to yield the [3,3,0]-bicyclic γ-lactone (±)-**81** in a high yield with a high diastereomeric ratio (9:1). After nine steps, the target molecule *salinosporamide A* (**82**) was achieved in a total of 15 steps with an overall yield of 6%. The cyclization step was deemed most crucial due to the necessity of obtaining the [3,3,0]-bicyclic γ-lactone with good diastereoselectivity (Scheme 20).

**Scheme 21:**
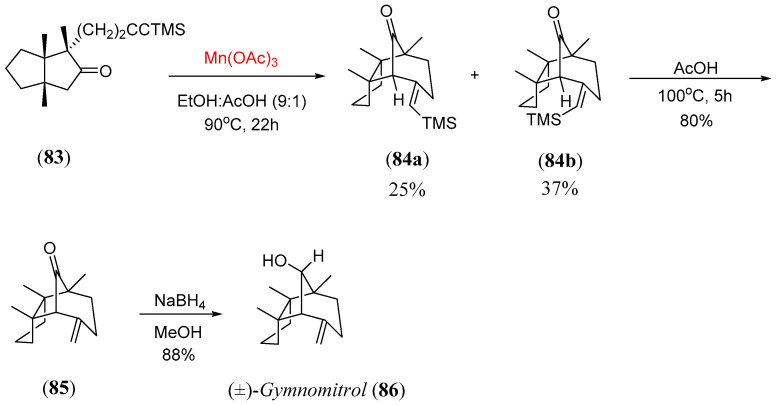
Synthesis of (±)-*gymnomitrol* (**86**).

O’Neil and Snider achieved the synthesis of the sesquiterpenoid compound, (±)-*gymnomitrol* (**86**), through a manganese(III)-mediated cyclization reaction. The synthesis commenced with the formation of an alkynyl ketone derivative of [(trimethylsilyl)butynyl] bicyclooctanone (**83**). Subsequently, manganese(III)-mediated radical generation facilitated the synthesis of *cis*- and *trans*-trimethylsilyl-substituted tricyclo[5.2.1]undecane-11-one (**84**), with yields of *cis*-**84a** at 25% and *trans*-**84b** at 37%. The hydrolysis of the trimethylsilyl group in refluxing AcOH, followed by the reduction of the keto group, resulted in the synthesis of (±)-*gymnomitrol* (**86**) with an overall yield of 16% (Scheme 21) [76].

**Scheme 22:**
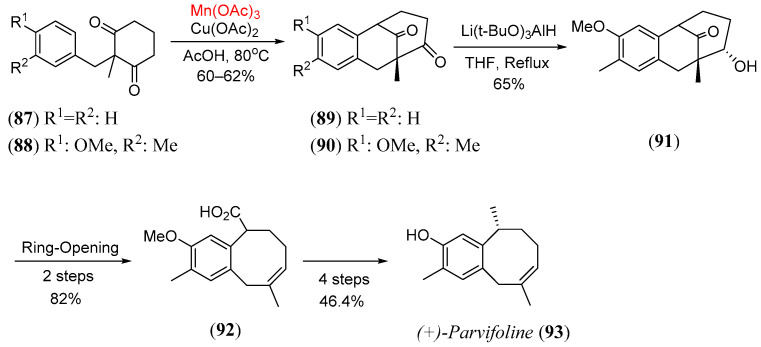
The synthesis of (+)-*parvifoline* (**93**).

Venkateswaran conducted the synthesis of the phenolic sesquiterpene (+)-*parvifoline* (**93**), achieving the formation of benzobicyclo[3,3,1]nonane using manganese(III) acetate, followed by the application of a ring-opening protocol (Scheme 22) [77]. Typically, the synthesis of this compound involves numerous steps. However, with the assistance of Mn(III), the desired bicyclic compound was obtained in a single step. Venkateswaran devised a meticulous synthetic strategy involving the ring-opening of the bicyclic compound (**91**) to yield benzocyclooctane (**92**), an analogue of (+)-*parvifoline* (**93**). The literature reports a total of 4 steps for the transformation of (**92**) to (**93**), achieving an overall yield of 46.4% [78].

**Scheme 23:**
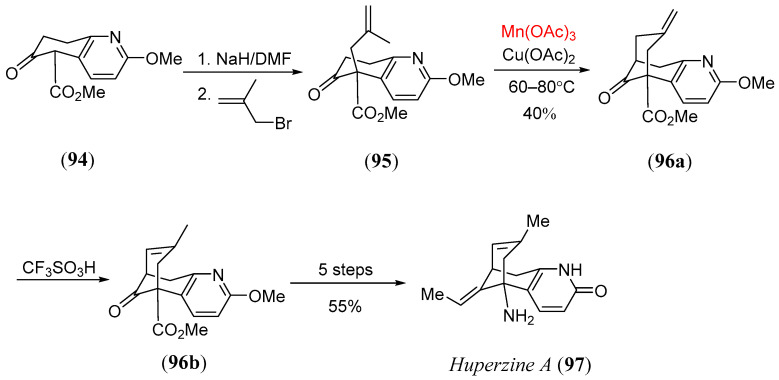
Synthesis of *Huperzine A* (**97**).

Lee and co-workers also reported the synthesis of *huperzine A* (**97**) [79]. This compound features a bicyclic skeleton fused with piperidine-2-one. To achieve this, β-ketoester (**94**) was alkylated with an appropriate allyl bromide to obtain the allyl product (**95**). The subsequent radical cyclization of (**95**) led to the formation of the corresponding *huperzine* analogue (**96**) (Scheme 23). Interestingly, the thermodynamically unstable *exo*-cyclization product (**96a**) of the *huperzine* intermediate easily isomerized to the endocyclic compound (**96b**), the other *huperzine* analogue. This compound was then converted to *Huperzine A* using the procedure in the literature, with a total yield of 22% [80].

**Scheme 24:**
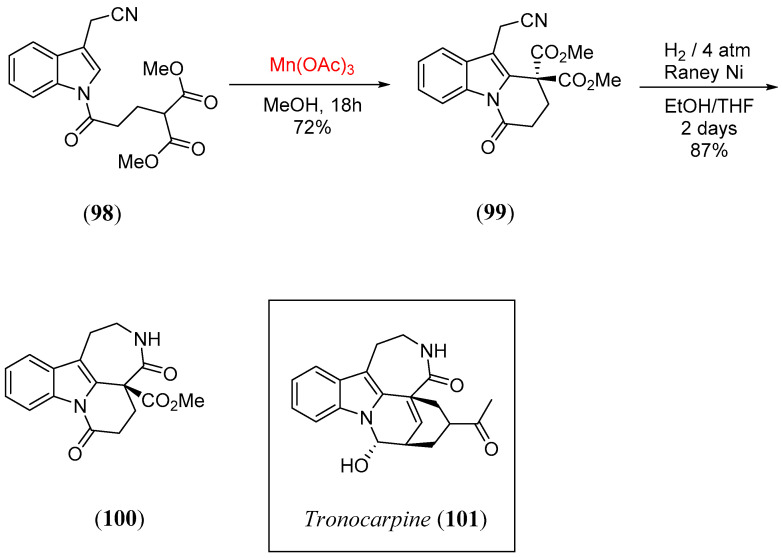
Synthesis of tetracyclic core of *tronocarpine* (**101**).

Magolan et al. designed a general and efficient strategy to identify the core structure of *tronocarpine*, which is particularly appealing. They synthesized a tetracyclic intermediate product with a similar skeleton to *tronocarpine* using manganese(III) acetate, starting with an N-substituted indole compound (**98**) (Scheme 24). The addition of a malonate radical, formed by manganese acetate, to the 2-position of the indole resulted in the formation of a tricyclic intermediate (**99**). The subsequent reduction of the cyano group to an amine, followed by cyclization with an ester, afforded the tetraycyclic core (**100**) of *tronocarpine* (**101**) [81].

**Scheme 25:**
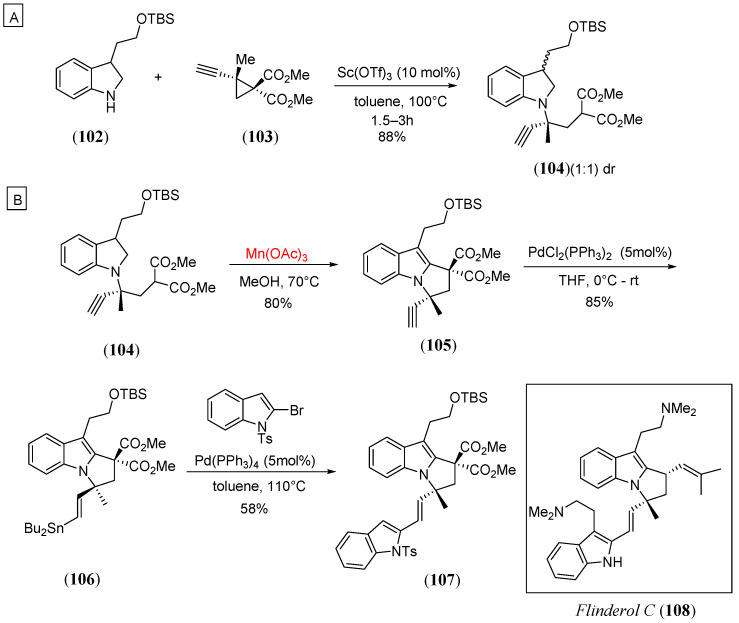
(**A**) Synthesis of (**104**) at initial step (**B**) Synthesis of *flinderol C* (**108**) by intramolecular radical addition reaction.

Tejeda et al. conducted a study involving the addition of a radical to indole, resulting in the synthesis of *flinderole C* (**108**) through radical intramolecular cyclization, with manganese(III) acetate serving as a radical initiator (Scheme 25). In the initial step, a N-substituted indoline compound (**104**) was formed by nucleophilic addition to cyclopropane derivatives, employing Yb(OTf)_3_ as a Lewis catalyst, followed by radical cyclization to produce 1,2-pyrroloindole (**105**). Following this pivotal step, hydrostannylation and Stille coupling reactions were employed to obtain the *flinderol C* substructure (**107**). The total synthesis yielded 32%, attributed to the efficiency of the overall synthetic process [82].

**Scheme 26:**
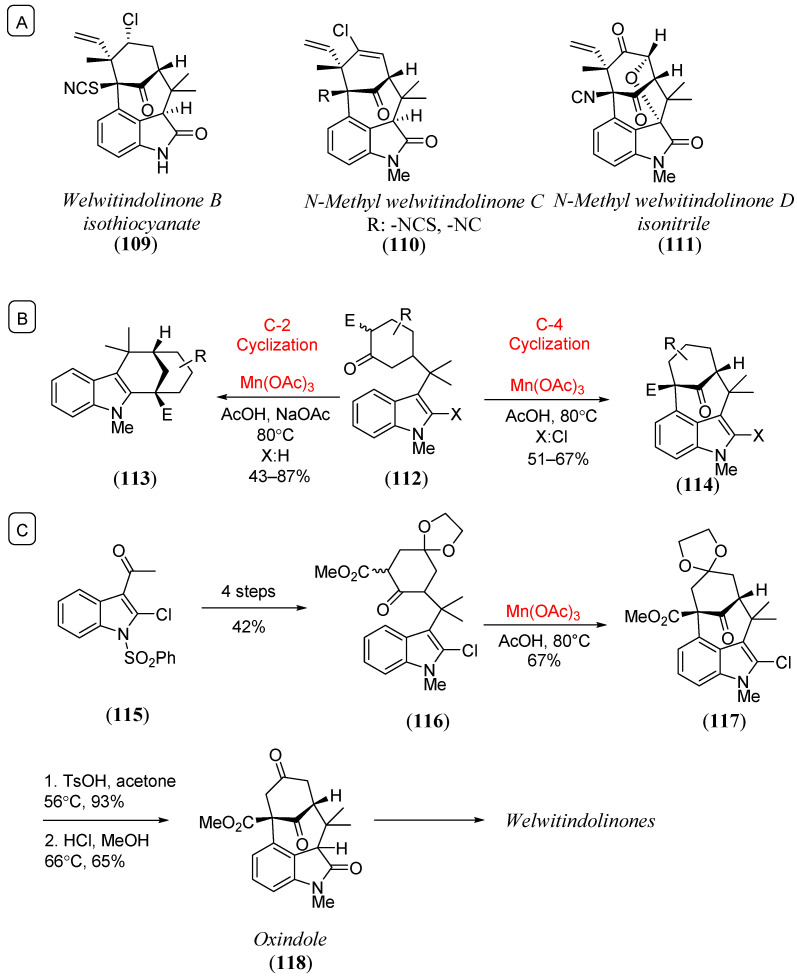
(**A**) The core structure of *welwitindolinone* alkaloids (**109, 110, 111**) (**B**) C-2 and C-4 cyclization by the assistance of Mn(OAc)_3_ (**C**) Synthesis of *oxindole* (**118**).

Bhat et al. reported the oxidative cyclization of three substituted indoles, aimed at constructing the core structure of welwitindolinone alkaloids, which possess a bicyclo[4.3.1]decane skeleton (Scheme 26A). The cyclization predominantly occurred at the C-4 position of the indole when the C-2 position was substituted with chloride, as illustrated in Scheme 26B. Conversely, when the C-2 position was unsubstituted, the cyclization pathway arose at the C-2 position. The synthesis was initiated with compound (**115**) to construct ketoester (**116**) (Scheme 26C). The key step involved the radical cyclization by manganese(III) acetate of (**116**) from the C-4 position, yielding the tetracyclic compound (**117**) with a high yield. Subsequently, *oxindole* (**118**) was formed to achieve the core structure of *welwitindolinone* alkaloids [83].

**Scheme 27:**
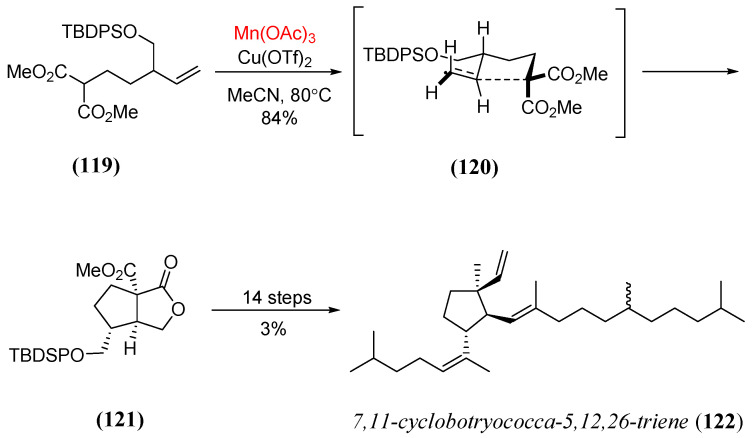
Synthesis of *7*,*11*-*cyclobotryococca*-*5*,*12*,*26*-*triene* (**122**) and the possible transition state of the radical addition reaction.

Davies et al. reported the synthesis of *7*,*11*-*cyclobotryococca*-*5*,*12*,*26*-*triene*, a *botryococcene* (**122**)-related natural compound. They initiated the synthesis by employing 4-pentenyl malonate (**119**) to obtain [3.3.0]-bicyclic γ-lactone (**121**) as a pivotal molecule, leading directly to the final molecule (Scheme 27). The [3.3.0]-bicyclic γ-lactone was obtained with a 75% yield and a 13:1 diastereomeric ratio. The radical addition underwent a 5-exo-trig cyclization with a chair-like transition state (**120**) [84,85].

**Scheme 28:**
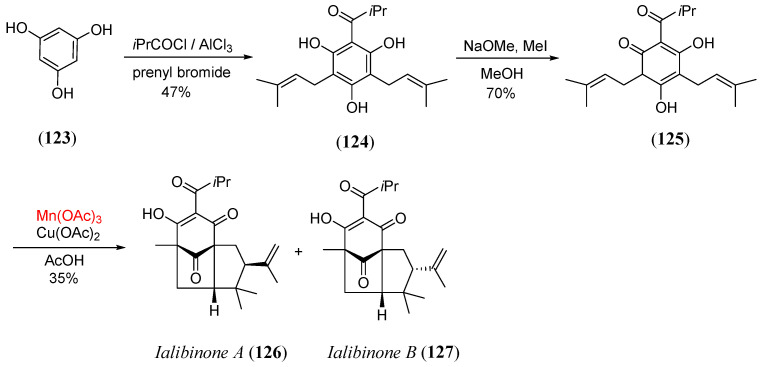
Synthesis of *ialibinone A* (**126**) and *ialibinone B* (**127**).

The synthesis of tricyclic natural compounds *ialibinone A* (**126**) and *ialibinone B* (**127**) (Scheme 28) was also developed by Simpkins and Weller using manganese(III) acetate [86]. The starting compound was commercially available *phloroglucinol* (**123**), which was subjected to Friedel–Craft to obtain the corresponding product (**124**) with unsaturated substituents. Then, to convert this substrate to (**125**) with an acidic proton, it underwent a radical cyclization with Mn(OAc)_3_ and Cu(OAc)_2_ to form target *ialibinone A* (**126**) and *ialibinone B* (**127**) products, respectively.

**Scheme 29:**
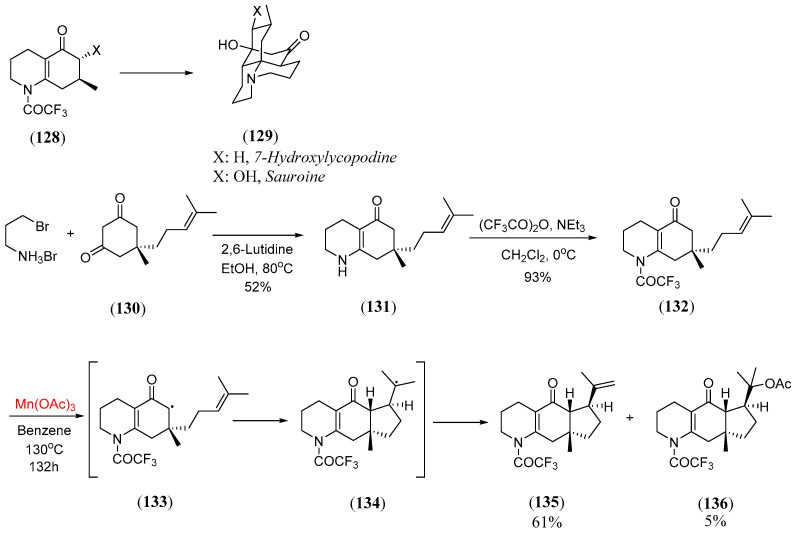
Synthesis of *sauroine* compounds.

Lin and Snider introduced an α’-acetoxidation method utilizing N-trifluoroacetyl-substituted vinylogous amide. They claimed that in the absence of a substituent on the nitrogen atom, instead of α’-acetoxidation, aromatization of the vinylogous amide to pyridine would occur (Scheme 29). To address this, the following methodology was employed: initially, the amide nitrogen was protected with a trifluoroacetyl group, followed by α’-acetoxidation with manganese(III) acetate to yield the acetoxidated compound. Utilizing this approach, they conducted a series of reactions to generate α’-carbon radicals, followed by addition to an alkene to obtain tricyclic derivatives (**135**) and (**136**), which represented the target *sauroine* compounds [87].

**Scheme 30:**
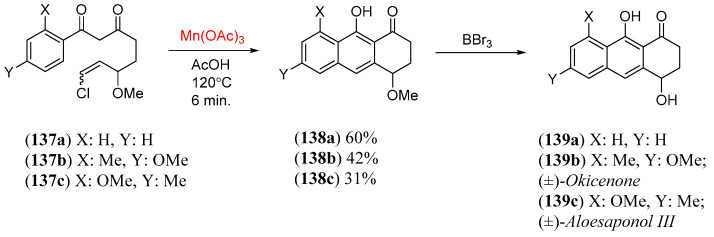
Synthesis of (±)-*okicenone* (**139b**) and (±)-*aloesaponol III* (**139c**).

Snider also accomplished the synthesis of (±)-*okicenone* (**139b**) and (±)-*aloesaponol III* (**139c**) through intramolecular tandem cyclization, with manganese(III) acetate serving as a crucial mediator (Scheme 30) [88]. The starting materials utilized were commercially available, and are as follows: 4-methoxy-2-methylacetophenone for (±)-*okicenone* (**139b**) and 2-methoxy-4-methylbenzoic acid for (±)-*aloesaponol III* (**139c**). Initially, a chloride-substituted enolizable compound was prepared to construct the tricyclic core for both *okicenone* and *aloesaponol III*. Chloride substitution in (**137**) was chosen for two reasons: firstly, to favor 6-exo-trig cyclization over 7-endo-trig due to steric hindrance posed by the presence of chloride, and secondly, to facilitate hydrogen chloride loss through the self-structuring to naphthol. Both natural products were synthesized via a three-step process to obtain racemic mixtures of (±)-*okicenone* (**139b**) and (±)-*aloesaponol III* (**139c**). The overall yields were calculated to be 17% and 9% for (**139b**) and (**139c**), respectively, starting from the aforementioned starting molecules. Notably, the radical cyclization reactions yielded 42% and 31% for (**139b**) and (**139c**), respectively.

**Scheme 31:**
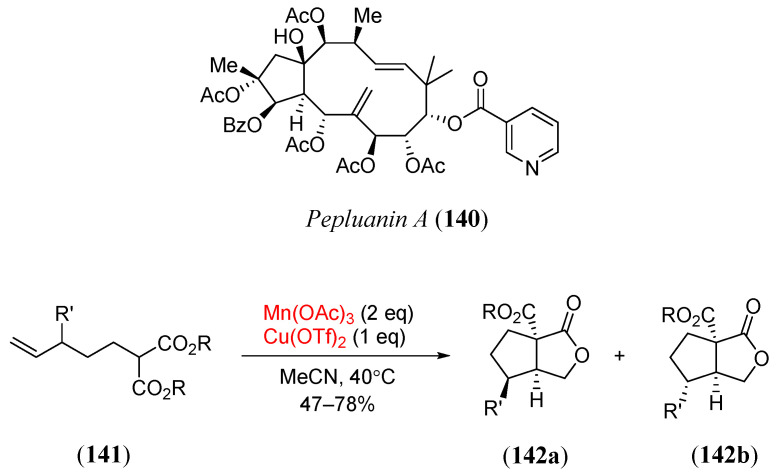
The synthesis *pepluanin A* derivatives.

**Scheme 32:**
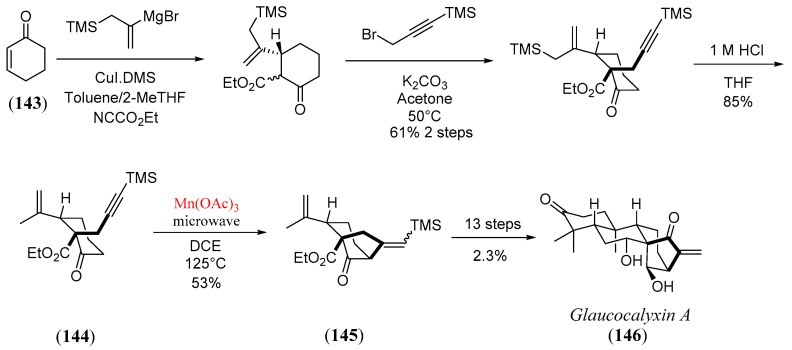
The synthesis of *glaucocalyxin A* (**146**).

Burton and colleagues reported the synthesis of *pepluanin A* (**154**) via the oxidative cyclization of pentenyl malonates, with manganese(III) acetate serving as the pivotal step to construct the cyclopentane skeleton. To accomplish this synthesis, they utilized t-butyl acetate and acrolein, followed by alkylation, reduction, and dicarboxylation to obtain the target molecule, a pentenyl malonate derivative (**141**). Subsequently, they explored the enantioselective synthesis of the cyclopentane core of *pepluanin A*, following the careful selection of protection groups in the molecule (Scheme 31). This work represents a significant advancement toward the synthesis of the *pepluanin A* natural compound [89].

(−)-*Glaucocalyxin A* (**146**), featuring a bicyclo[3,2,1]octane with a three-ring system, was successfully synthesized by Guo et al. via radical cyclization starting from a commercially available compound (**143**). The key radical cyclization reaction is illustrated in Scheme 32. Following the formation of a carbon radical mediated by manganese(III), an addition to alkyne (**144**) was implemented to construct the bicyclo[3,2,1]octane core (**145**), identified as the pivotal step in the synthesis of (−)-*glaucocalyxin A*. Subsequently, after obtaining (**145**), 13 additional steps were applied, resulting in an overall yield of 2.3%. Notably, unlike other manganese(III)-mediated radical cyclization reactions, particularly in natural product synthesis, a microwave synthesis was reported to afford a 53% yield [90].

**Scheme 33:**
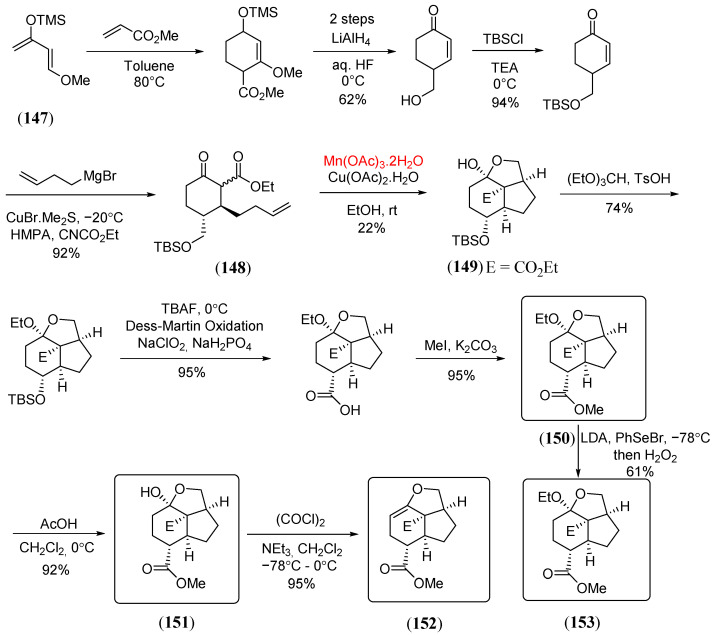
The synthesis of *picrotoxane* derivatives (**151**, **152**, **153**).

*Picrotoxane*, a sesquiterpene alkaloid group of compounds, was synthesized by Cao et al. The key step involved a radical cyclization reaction to construct the tricyclic compound (**149**) mediated by manganese(III) acetate, resulting in the formation of *picrotoxane* derivatives, as illustrated in Scheme 33. The overall yield for (**151**) was 7.2%, while (**152**) and (**153**) were found to be 6.9% and 7.9%, respectively. Additionally, 5-exo radical cyclization intermediates were proposed, and DFT calculations were reported according to the cation intermediate mechanism [91].

**Scheme 34:**
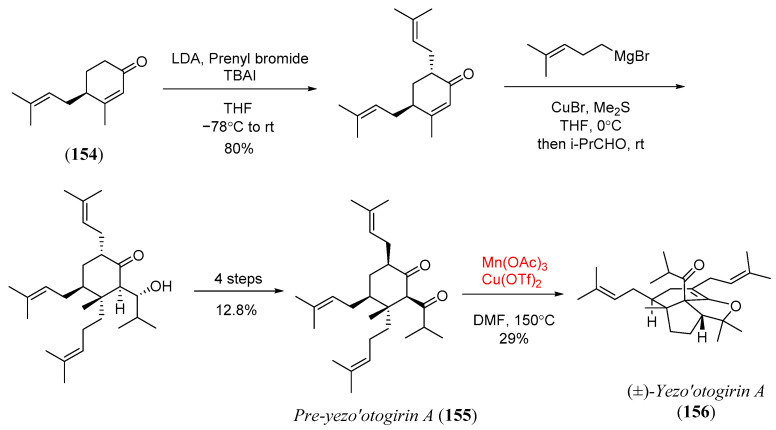
The synthesis of (±)-*yezo’otogirin A* (**156**).

Lam et al. reported the synthesis of a (±)-*yezo’otogirin A* natural product starting from commercially available material (**154**), which was obtained in two steps [92]. The key step involved a radical cyclization reaction initiated by manganese(III) acetate, allowing for the efficient preparation of the *yezo’otogirin* ring system. This synthesis was achieved in conjunction with Cu(OTf)_2_ as the co-oxidant agent. The entire synthetic pathway comprised nine steps with an overall yield of 3%, while the radical cyclization step alone yielded 29%. To initiate the successful synthesis of (±)-*yezo’otogirin A* (**156**), a β-keto ester *pre*-*yezo’otogirin* (**155**) was initially obtained (Scheme 34) [93].

**Scheme 35:**
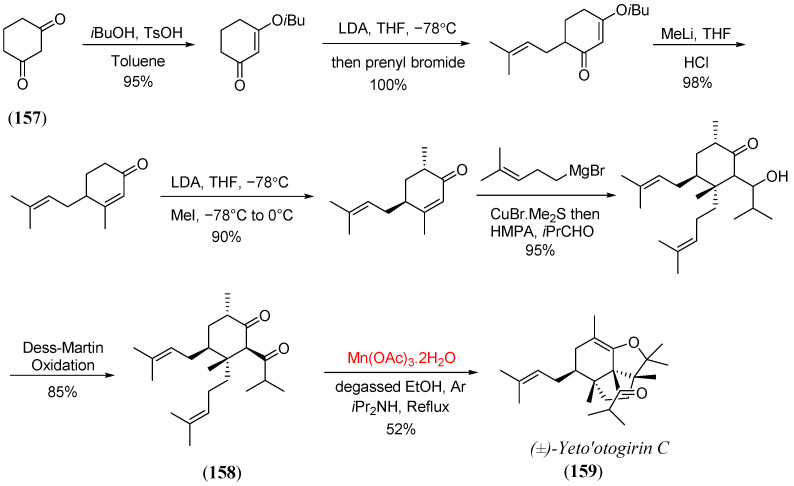
The synthesis of (±)-*yeto’otogirin C* (**159**).

He et al. synthesized the *yezo’otogirin* natural product (±)-*yeto’otogirin C*, employing a different approach compared to other radical cyclization reactions. In this method, manganese(III) was utilized along with oxygen for the in situ production of manganese(III). Similar to the synthesis of (±)-*yeto’otogirin A*, this process also employed the same precursor, β-keto ester (**158**), in the radical cyclization step. This precursor was synthesized from cyclohexanedione (**157**). The entire synthesis of (±)-*yeto’otogirin C* (**159**) was achieved with an overall yield of 31% and involved four steps (Scheme 35) [94,95].

**Scheme 36:**
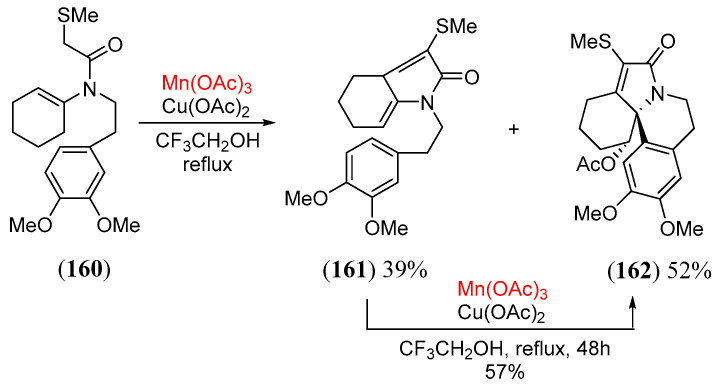
Synthesis of *erythrinane* (**162**).

Toyao and Chikaoka et al. presented an intriguing study on the synthesis of *erythrinane* (**162**) via a radical intramolecular reaction, starting from α-methylthioamides (**160**), as illustrated in Scheme 36. Initially, cyclization occurs to obtain (**161**), followed by further oxidation with Mn(OAc)_3_/Cu(OAc)_2_ in CF_3_CH_2_OH under reflux, resulting in the formation of the target *erythrinane* (**162**) [96,97].

**Scheme 37:**
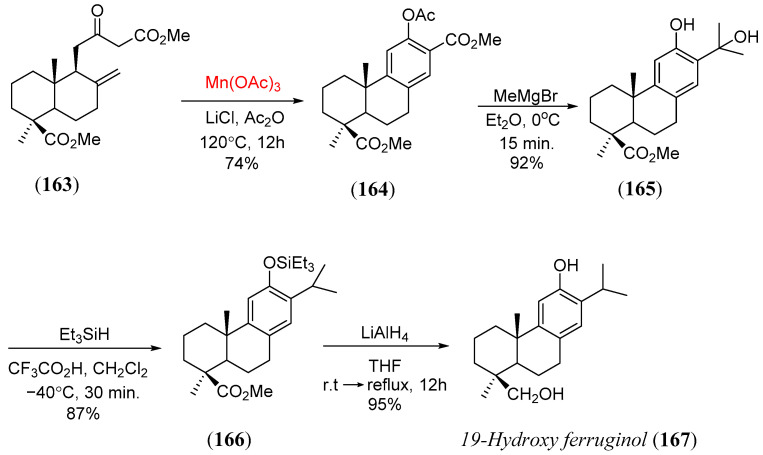
Synthesis of diterpene compound abietane *derivative 19*-*hydroxy ferruginol* (**167**) by radical addition reaction.

The synthesis of an *abietane* derivative diterpene compound was reported by Alvarez-Manzaneda. This synthetic pathway entails multi-step reactions, with the pivotal stage involving the utilization of manganese(III) for cyclization. The reaction sequence is depicted in Scheme 37. Initially, the reaction of β-ketoester (**163**) with a methylene group facilitates cyclization to yield (**164**) [98]. Subsequently, the addition of a methyl group results in (**165**) via a Grignard reaction, followed by Silyl protection to enable the reduction of the methyl ester group of (**166**). Upon reduction with LiAlH_4_, the desired diterpene compound, *19*-*hydroxy ferruginol* (**167**), was produced.

**Scheme 38:**
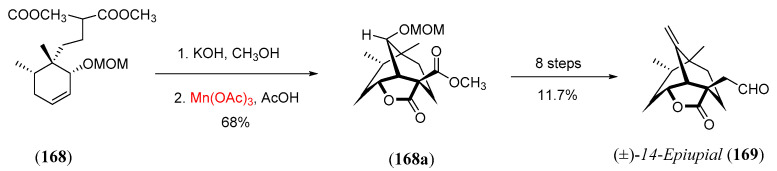
Synthesis of (±)-*14*-*epiupial* (**169**).

**Scheme 39:**
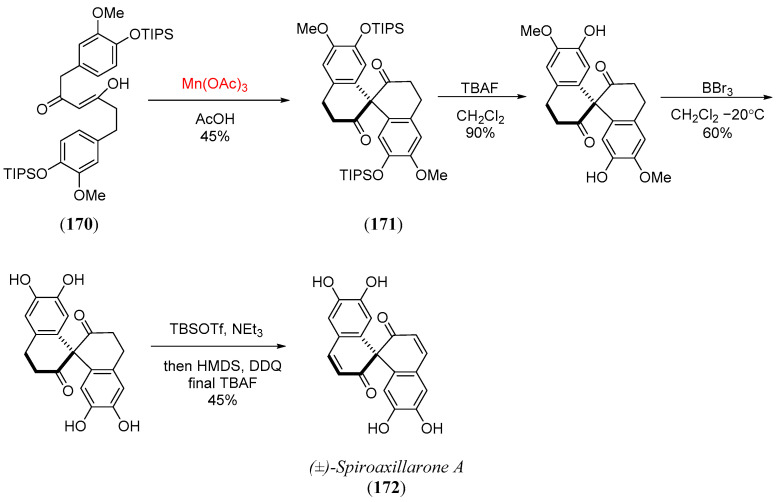
The synthesis of (±)-*spiroaxillarone A* (**172**) natural product.

Paquette and colleagues have detailed a concise pathway for synthesizing (±)-*14*-*epiupial* (**169**) utilizing manganese(III) acetate chemistry, with credit given to lactonization in a multi-step synthesis process (Scheme 38). It is observed that only one epimer undergoes cyclization, yielding the corresponding lactone (**168a**) stereochemically [99]. The pivotal stage entails lactonization mediated by manganese(III) acetate, yielding a 68% yield. Following the synthesis of lactone (**168a**), (±)-*14*-*epiupial* (**169**) was obtained through an additional five steps. Alternatively, Takahashi conducted a comparative study on the synthesis of (+)-*upial*, an analogue of (±)-*14*-*epiupial*, in 15 steps, achieving a total yield of 10% [100].

(±)-*Spiroaxillarone A* belongs to the spirobisnaphthalene compound class, and its synthesis was accomplished through a strategic one-step cycloaddition–oxidation reaction starting from a *curcumin* (**170**) derivative. Following the formation of the corresponding spirobisnaphthalene derivative (**171**), demethylation leads to the production of (±)-*spiroaxillarone A* (**172**) with an overall yield of 11%. This procedure exemplifies the efficacy of manganese(III) acetate-based radical cyclization reactions when employing an appropriate starting material to obtain the desired natural compounds (Scheme 39) [101].

**Scheme 40:**
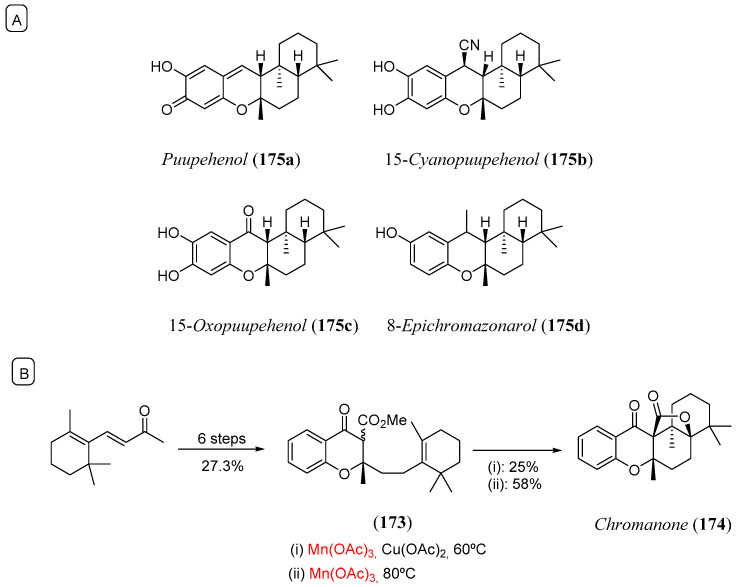
(**A**) The sesquiterpene–phenol skeletons (**B**) Free radical cyclization usage for the synthesis of sesquiterpene-phenol natural products (**175**).

The sesquiterpene–phenol skeleton serves as the primary framework for various natural products. Crombie et al. synthesized chromanone (**174**) as a precursor for the production of *puupehenol* (**175a**), *15*-*cyanopuupehenol* (**175b**), *15*-*oxopuupehenol* (**175c**), and *8*-*epichromazonarol* (**175d**), compounds renowned for their potential therapeutic effects. Notably, the synthesis of the pivotal compound, chromanone (**174**), was accomplished in four steps, with the final step involving free radical cyclization facilitated by manganese(III) acetate. However, two methods were explored to obtain compound (**174**). In the initial approach, copper(II) acetate, in combination with manganese(III) acetate, was utilized at 60 °C, yielding a 25% yield. Conversely, employing only manganese(III) acetate resulted in a 58% yield at 80 °C (Scheme 40) [102]. Once (**174**) was obtained, the subsequent production of target natural products became straightforward, given that the fundamental skeleton was constructed via radical cyclization.

### 3.2. Tandem Cyclizations

**Scheme 41:**
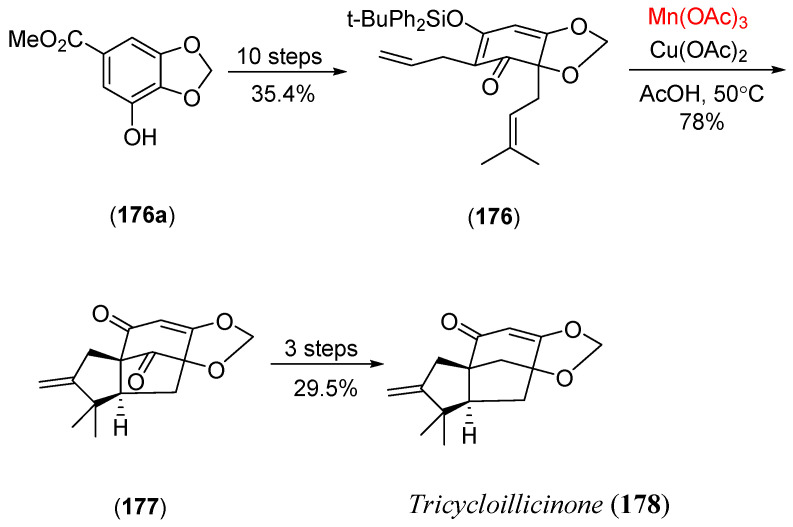
A tandem cyclization reaction mediated by manganese(III) and copper(II) acetate to obtain *tricycloillicinone* (**178**).

Pettus and his colleagues successfully synthesized *tricycloillicinone* (**178**) derivatives by employing tandem cyclization mediated by manganese(III) acetate as a crucial step to generate a radical adduct (**177**) (Scheme 41). In this procedure, a β-diketone (**176**) underwent a tandem cyclization reaction, yielding (**177**) with an impressive yield of 78%, which is notably high for reactions of this type. It has been reported that the cyclization reaction occurs subsequent to the cleavage of the silyl enol ether, leading to the formation of the β-diketone. The overall yield was reported to be 8.1% [103].

**Scheme 42:**
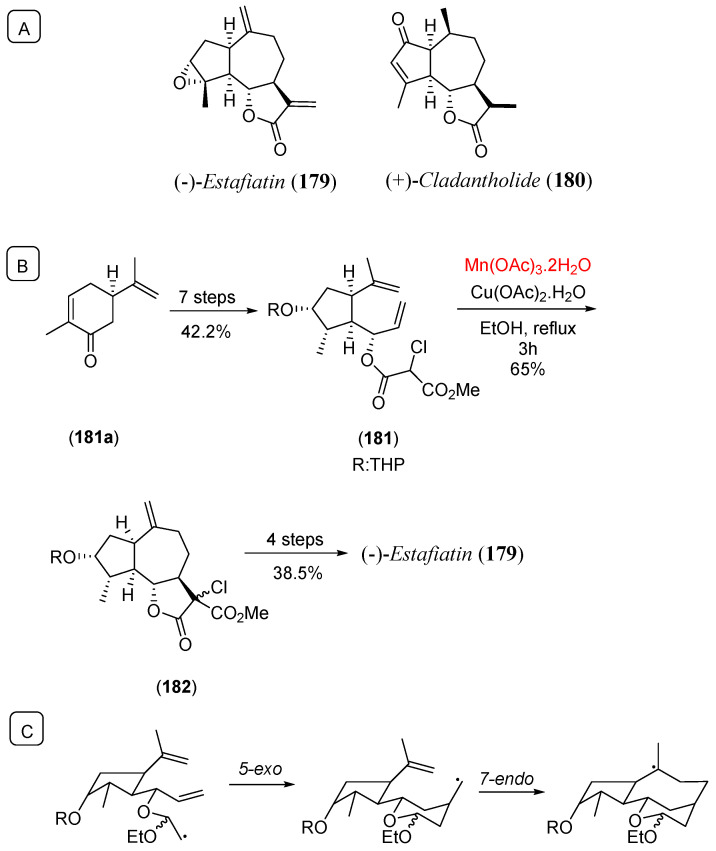
(**A**) (−)-*estafiatin* (**179**) and (+)-*cladantholide* (**180**) (**B**) synthesis of (**179**) and (**180**) and (**C**) tandem radical cyclization steps.

Lee and his co-workers documented a total synthesis of (−)-*estafiatin* (**179**) and (+)-*cladantholide* (**180**), sesquiterpene lactones, as outlined in Scheme 42A. The formation of the seven-membered ring (**182**) involved a tandem cyclization of dialkene (**181**), proceeding from a 5-*exo* lactonization, followed by a 7-*endo* cyclization (Scheme 42B,C). In addition to manganese(III) acetate, copper(II) acetate was employed to facilitate radical cyclization, yielding a diastereomeric ratio of 3:1. The reported yield for this pivotal step was 65%, representing a critical advancement towards efficient product synthesis [104].

**Scheme 43:**
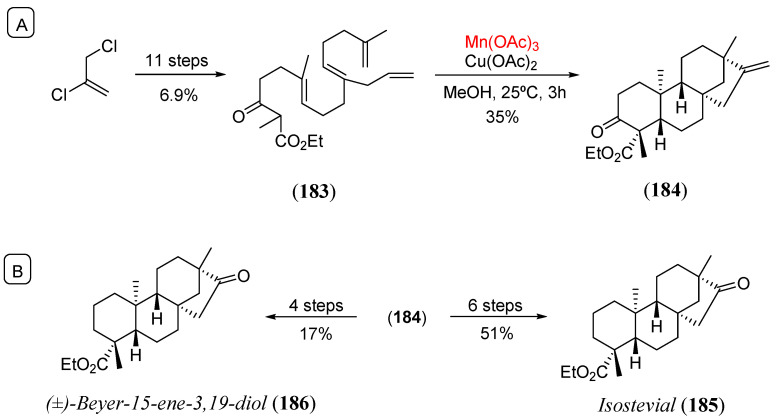
(**A**) Synthesis of precursor (**184**) and (**B**) synthesis of *isostevial* (**185**) and (±)-*beyer*-*15*-*ene*-*3β*,*19*-*diol* (**186**) from (**184**).

A tandem cyclization reaction mediated with manganese(III) for the synthesis of (±)-*isostevial* (**185**) and (±)-*beyer*-*15*-*ene*-*3β*,*19*-*diol* (**186**) natural compounds was achieved by Snider (Scheme 43). Upon this synthesis, radical tandem cyclization occurred in the first step to obtain a tetracyclic compound (**184**), which was a key step. After deriving the core skeleton, total syntheses of the final natural products (±)-*isostevial* (**185**) and (±)-*beyer*-*15*-*ene*-*3β*,*19*-*diol* (**186**) were carried out in six and four steps to reach 51% and 17% yields, respectively [105].

**Scheme 44:**
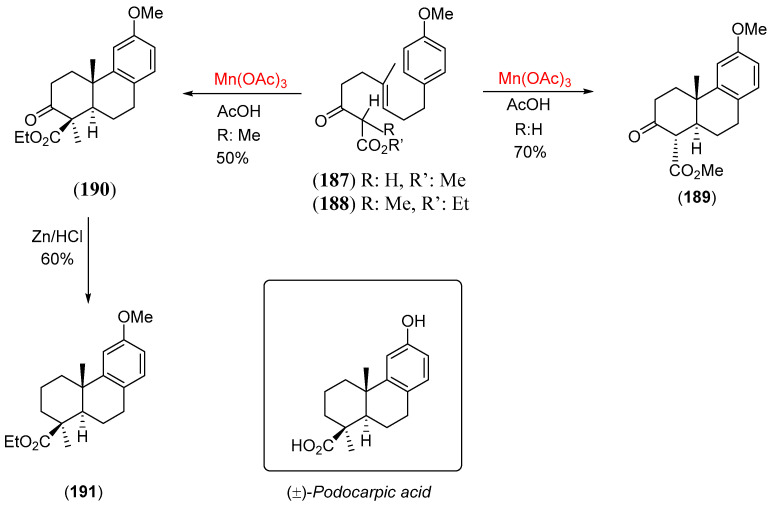
Synthesis of (±)-*podocarpic acid* analogue (**191**).

Snider also presented a synthetic pathway for the production of (±)-*podocarpic acid* analogue (**191**) through the application of free radical chemistry mediated by manganese(III) acetate (Scheme 44). The pivotal stage involved intramolecular tandem cyclization to form a fused tricyclic intermediate (**190**). Following this, Clemmensen reduction using Zn/HCl was conducted to yield the corresponding product, (±)-*podocarpic acid* analogue (**191**) [106].

**Scheme 45:**
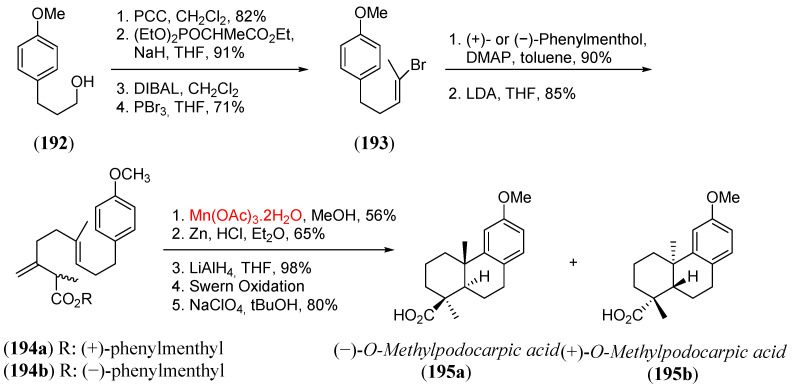
Synthesis of (−)-*O*-*methylpodocarpic acid* (**195a**) and (+)-*O*-*methylpodocarpic acid* (**195b**).

Zhang and Snider also detailed the synthesis method for (+)-*O*-*methylpodocarpic acid* (**195b**) and (−)-*O*-*methylpodocarpic acid* (**195a**) with highly optimized stereoselectivity (Scheme 45). Beginning with alcohol (**192**), a terminal alkene (**193**) was initially obtained. To achieve the desired stereoselectivity, (+) and (−)-phenylmenthol were employed separately to form (**194**) in distinct reactions. For the synthesis of (−)-*O*-*methylpodocarpic acid* (**195b**), the (−)-phenylmenthol isomer of (**194b**) was prepared. Utilizing a tandem cyclization of this isomer, tricyclic diastereomers were obtained with a 50% yield. Subsequently, after four subsequent steps, (−)-*O*-*methylpodocarpic acid* (**195b**) was derived. Conversely, for the synthesis of (+)-*O*-*methylpodocarpic acid* (**195a**), the (+)-phenylmenthol isomer of (**194a**) was utilized [107].

**Scheme 46:**
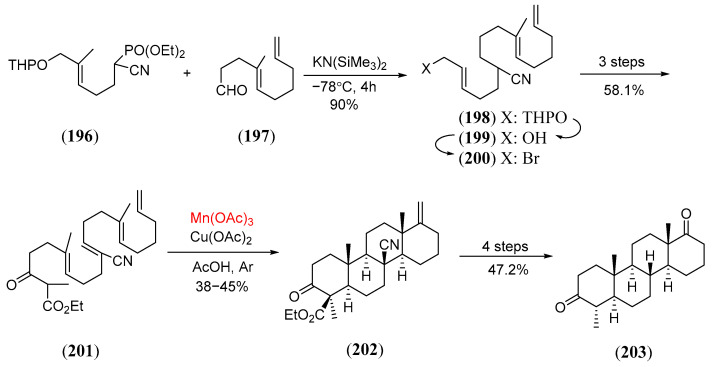
The key step in the synthesis of *D*-*homoandrostane* derivative (**203**).

Zoretic et al. presented a synthetic approach to produce *d*,*l*-*5α*-*D*-*homoandrostane*-*3*,*17a*-*dione* (**203**). The pivotal step involved a tandem cyclization of tetraene (**201**) by inducing radical formation on the methyl β-keto ester, resulting in the formation of the tetracyclic compound (**202**) (Scheme 46). Following this cyclization step, additional carefully designed stereoselective methods were employed to attain the desired steroid compound (**203**) [108].

**Scheme 47:**
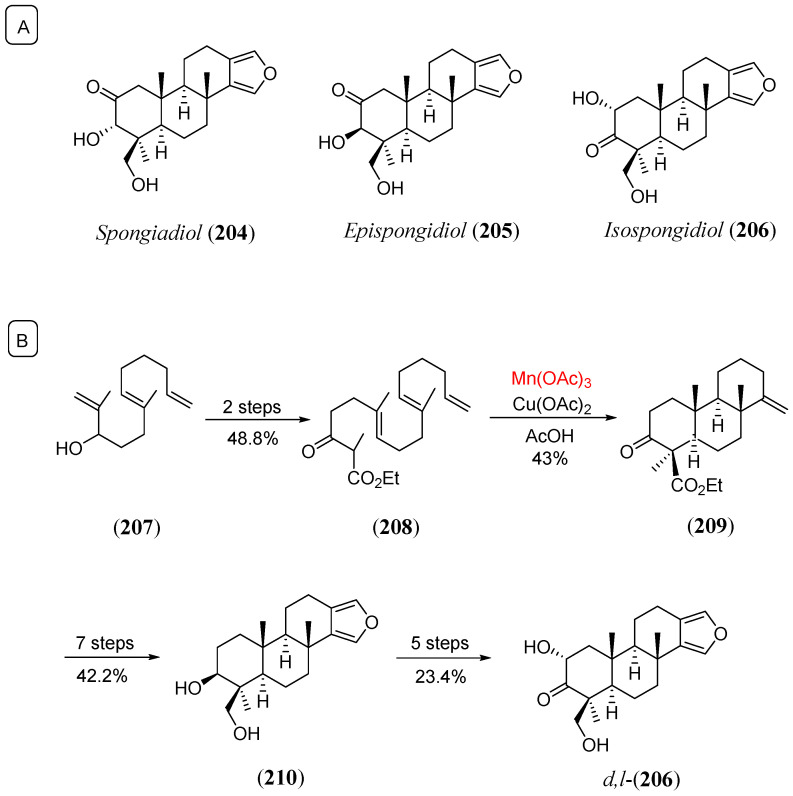
(**A**) Furanoditerpene derivatives, and (**B**) the synthesis of *spongian* skeleton (**210**).

*Spongiadiol* (**204**), *epispongidiol* (**205**) and *isospongidiol* (**206**) compounds are members of the furanoditerpene class, and were isolated from a marine sponge *Spongia Linnaeus* (Scheme 47A). The most significant part of the synthesis of these compounds is the formation of tricyclic ring. Zoretic and co-workers suggested tandem cyclization to form tricyclic ring starting from an appropriate isolated triene compound (**207**) (Scheme 47B). After deriving the tricyclic skeleton (**209**) by a tandem cyclization of (**208**) via manganese(III), a multi-step reaction was conducted to obtain a *spongian* skeleton (**210**) in seven steps, reaching a 42.2% total yield. Having obtained (**210**), they carried on the synthesis of *isospongiadiaol*, thus achieving a racemic *d*,*l*-*isospongiadiol* (**206**) mixture with five additional steps [109].

**Scheme 48:**
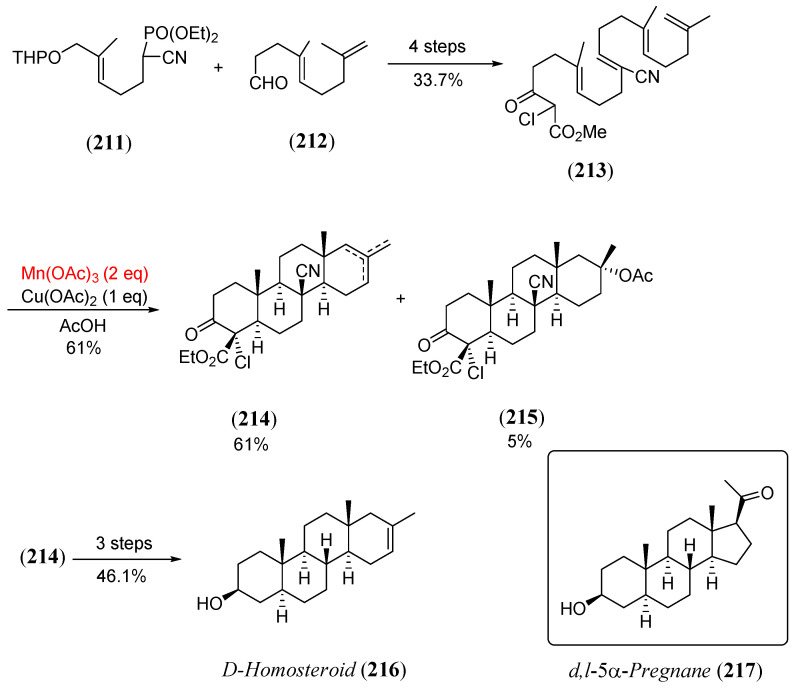
Tandem cyclization reaction of *d*,*l*-*5α*-*pregnane* (**217**).

Zoretic et al. achieved a synthetic pathway for the production of *d*,*l*-*5α*-*pregnane* steroid analogue *D*-*homosteroid* (**216**) via a tandem cyclization reaction (Scheme 48). Initially, they commenced the synthesis by constructing the key compound triene (**213**) through a three-step process. An oxidative tandem cyclization of triene (**213**) yielded the corresponding tetracyclic isomer (**214**) with a yield of 61%, along with the tetracyclic acetate derivative (**215**) in a 5% yield. Following the acquisition of tetracyclic (**214**), they transformed it into the precursor of *5α*-*pregnanes*, *D*-*homosteroid* (**216**), through a three-step sequence, achieving a total yield of 46% [110].

**Scheme 49:**
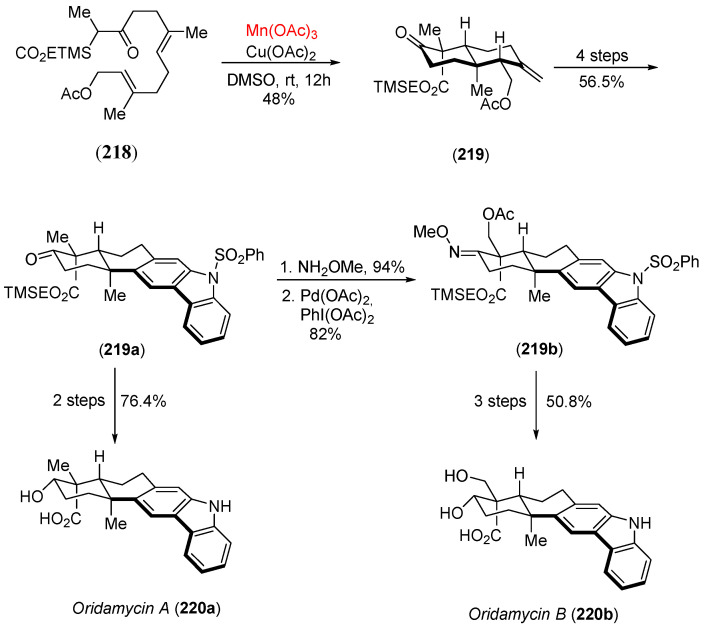
Synthesis of *oridamycin* family with manganese(III) acetate as a key step.

Meng reported a comprehensive synthesis study showcasing a tandem cyclization of a β-keto ester (**218**) to yield a single diastereomer trans-decalin (**219**) with a 48% yield, facilitated by Cu(OAc)_2_ in DMSO (Scheme 49). Subsequently, in the following reactions, *oridamcin A* and *oridamycin B* were synthesized. Decalin (**219**) played a crucial role as a key intermediate, enabling the entire reaction process, as the primary skeleton of *oridamycin* (**220**) was formed through free radical tandem cyclization [111]. Similarly, Trotta also presented a comparable study detailing the production of *oridamycin A* (**220a**) and *oridamycin B* (**220b**) by synthesizing trans-decalin using manganese(III) acetate [112].

**Scheme 50:**
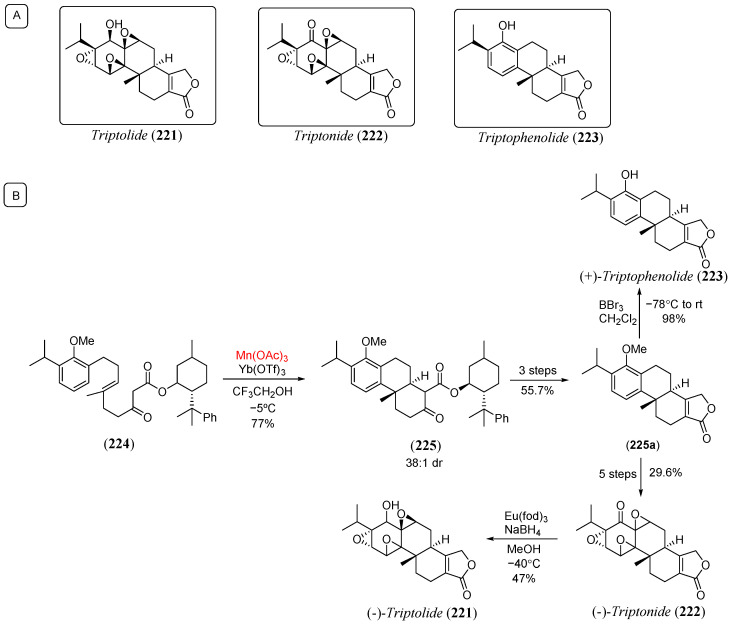
(**A**) Synthesis of (−)-*triptolide* (**221**), (−)-*triptonide* (**222**), and (+)-*triptophenolide* (**223**) compounds (**B**) Synthesis of (**221**), (**222**) and (**223**) by using Mn(OAc)_3_.

Yang and colleagues pursued the synthesis of (−)-*triptolide* (**221**), (−)-*triptonide* (**222**), and (+)-*triptophenolide* (**223**) utilizing manganese(III) acetate chemistry (Scheme 50). Additionally, they revealed the impact of lanthanide triflates as chelating agents to enhance stereoselectivity [113,114]. Their study involved the utilization of a range of lanthanide triflates such as Yb(OTf)_3_, Er(OTf)_3_, Sm(OTf)_3_, Y(OTf)_3_, and Pr(OTf)_3_ Lewis acids, which not only accelerated the reaction but also provided improved stereoselectivity with higher yields. The effect of lanthanide triflates was attributed to the coordination of bidentate carbonyl groups in the radical compound formed in the intermediate structure. This coordination facilitated radical addition from one side, thereby enhancing diastereoselectivity. Furthermore, to achieve the enantioselective synthesis of the target molecules, Yang employed (−)-8-phenylmenthol. The tandem oxidative cyclization of (+)-8-phenylmethylester (**224**) resulted in the formation of (**225**) with a 38:1 diastereomeric ratio, followed by the transformation of (**225**) to (**225a**) in three steps with a yield of 55.7%. The demethylation of (**225a**) readily furnished (+)-*triptophenolide* (**223**) in a 98% yield [115].

**Scheme 51:**
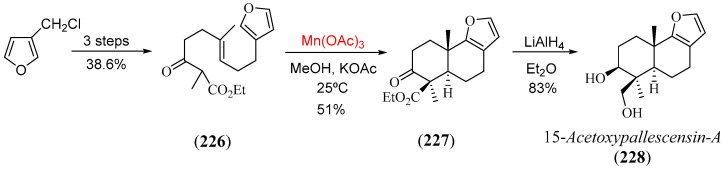
Synthesis of *15*-*acetoxypallescensin*-*A* (**228**).

Snider et al. reported the synthesis of *15*-*acetoxypallescensin*-*A* (**228**) in just five steps, starting from commercially available 3-chloromethylfuran, achieving an overall yield of 16.3% [116]. In Scheme 51, the reaction proceeds through an intramolecular tandem cyclization, with manganese(III)-assisted radical cyclization being the pivotal step. When compared to the study by Zoretic, which involved the synthesis of *15*-*acetoxypallescensin*-*A* in 12 steps with an overall yield of 1.3%, Snider’s approach not only reduced the number of synthesis steps, but also resulted in an increased overall yield [117].

**Scheme 52:**
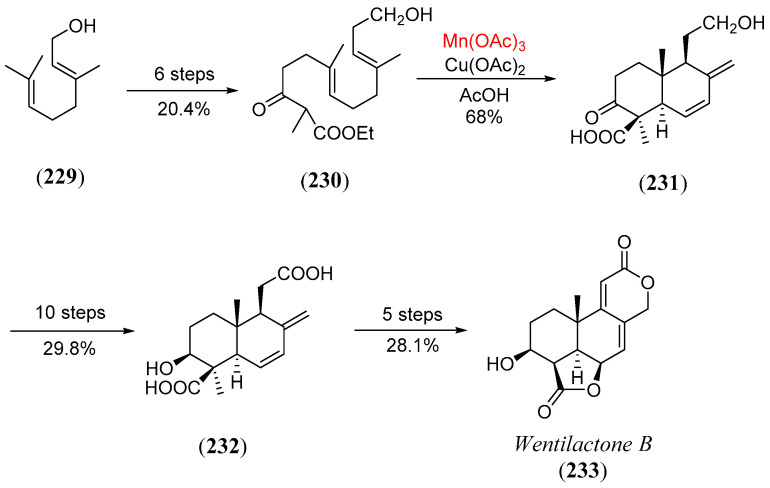
*Wentilactone B* (**233**) synthesis by tandem cyclization.

**Scheme 53:**
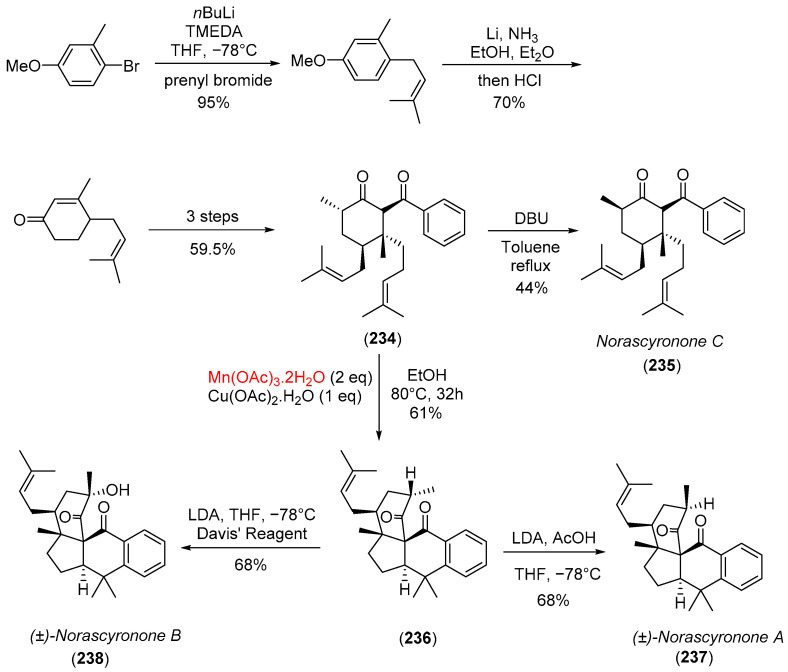
The synthesis of (±)-*norascyronones A* (**237**) and (±)-*norascyronones B* (**238**).

Barraro and his research group successfully synthesized the bicyclic core structure of *wentilactone B* (**231**), utilizing a manganese(III)-induced tandem cyclization procedure, followed by the formation of the tetracyclic *podolactone* skeleton via Pd(II) bislactonization (Scheme 52). Commencing from commercially available *geraniol* (**229**), a 3.5% yield was achieved for the synthesis of (**231**), with a particularly notable tandem cyclization yield of 68% facilitated by Mn(OAc)_3_. The total synthesis yield was calculated to be 0.3% [118].

(±)-*Norascyronones A* (**237**) and (±)-*norascyronones B* (**238**) contain four condensed cyclic structures with a five-stereogenic center. Cao et al. reported the synthesis of these natural products by radical cyclization via manganese(III) acetate. To achieve the production, they began with the synthesis of diketone (**234**) at first (Scheme 53). After deriving the appropriate starting compound diketone with an overall yield of 17.4% in three steps, a tandem cyclization was applied to derive (**236**) from Mn(OAc)_3_/Cu(OAc)_2_ in EtOH. This step was mentioned as the key step for the synthesis, and afterwards (±)-*norascyronones A* (**237**) and (±)-*norascyronones B* (**238**) were obtained in seven steps with a 16.4% overall yield. Also note that *norascyronones C* (**235**) was obtained from diketone (**234)** in a 44% yield [119].

**Scheme 54:**
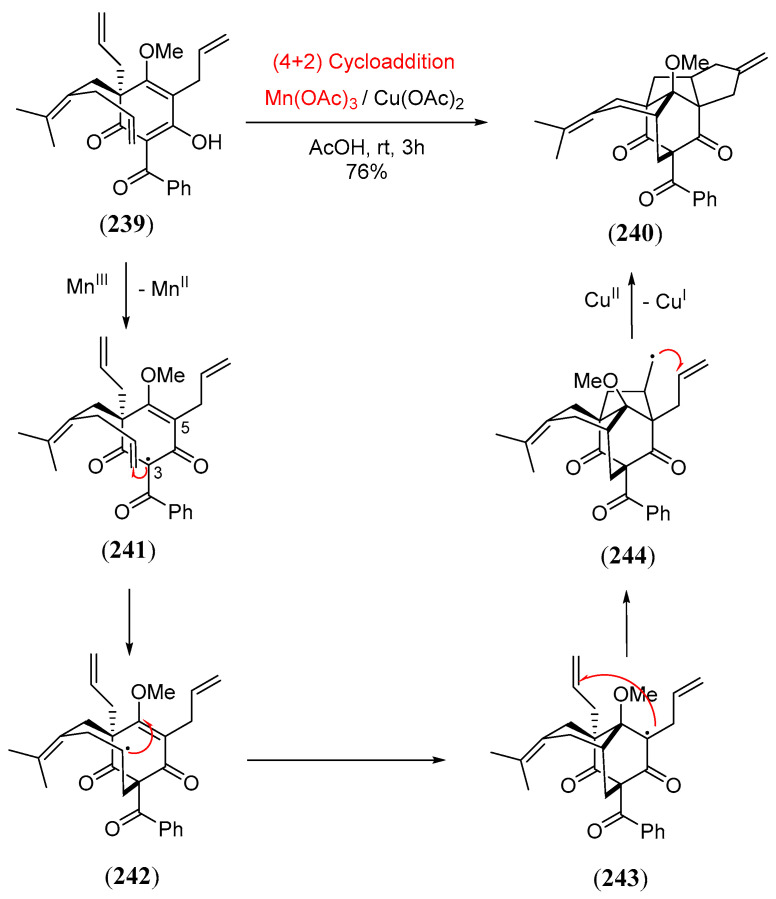
Formation mechanism of (**240**).

Finally, last but not the least, Mitasev et al. employed *phloroglucinol* substrates in oxidative [4 + 2] cycloaddition reactions via tandem cyclization mediated by manganese(III) chemistry [39]. The conversion mechanism is elucidated in Scheme 54. Despite its apparent complexity, a detailed review reveals that all reactions involve radical additions to alkenes to form cyclizations. In the initial step, a *phloroglucinol* derivative (**239**) forms a manganese(III)-enol structure (**241**), where a radical is generated on C-3, followed by addition to the closest alkene to construct (**242**). Subsequently, the radical intermediate again attacks the alkene to form the C-5 radical adduct (**243**). This C-5 radical then forms a pentacyclic intermediate (**244**), followed by radical addition to the allyl group and reaction with Cu(II), ultimately resulting in the loss of Cu(I) to construct the final compound (**240**). Upon overall investigation of the reaction, a [4 + 2] cycloaddition and 5-*exo* radical cyclizations were observed in the final product, with a yield of 76%.

### 3.3. Intramolecular Rearrangement Reactions

**Scheme 55:**
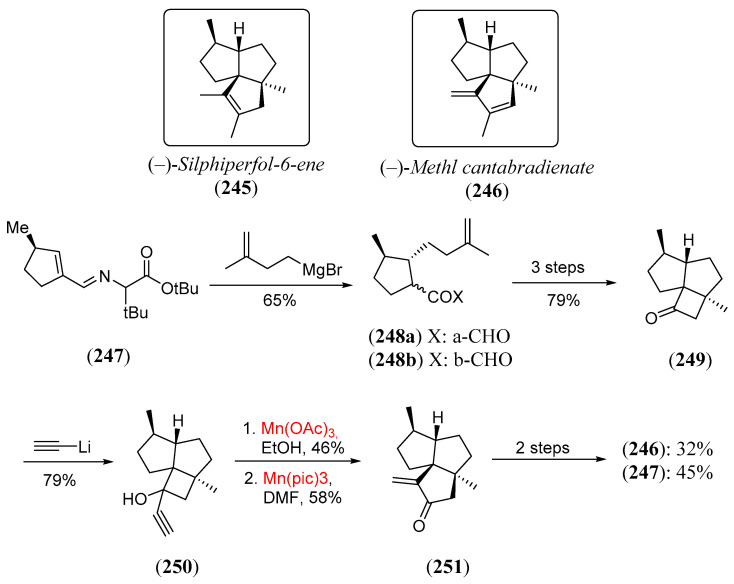
Synthesis of natural products by manganese(III) radical chemistry.

Snider synthesized (−)-*silphiperfol* (**245**) and (−)-*methyl cantabradienate* (**246**) (Scheme 55) in seven steps starting from (R)-3-methyl-1-cyclopentenecarboxyaldehyde, achieving overall yields of 13% and 9%, respectively [120]. The key step in the synthesis of these natural products was the utilization of manganese(III)-based cyclization. Snider employed a ring expansion of cyclobutane via oxidative radical rearrangement to form the target natural products. Initially, Snider prepared a cyclobutane derivative (**249**), and subsequently, by adding lithium acetylide to this compound, ethynyl cyclobutanol (**250**) was obtained with a yield of 79%. Treating this compound with Mn(pic)_3_ resulted in the formation of the key intermediate (**251**) with a yield of 58% (it was also noted that Mn(OAc)_3_ in EtOH gave a yield of 46%). The formed methylene cyclopentanone (**251**) was then converted into (**245**) and (**246**) with appropriate reagents in two steps. In conclusion, (**245**) and (**246**) were successfully synthesized by intramolecular [2 + 2] ketene cycloaddition followed by manganese(III)-mediated oxidative radical rearrangement.

**Scheme 56:**
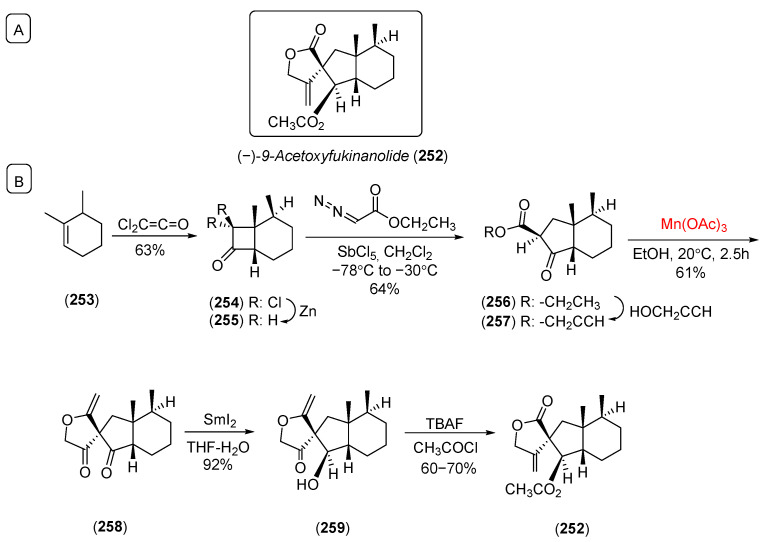
(**A**) Molecular structure of (−)-*9*-*acetoxyfukinanolide* (**252**) and (**B**) the synthesis steps.

Hamelin et al. reported an efficient synthetic route for the synthesis of (−)-*9*-*acetoxyfukinanolide* (**252**), a member of the *bakkane* family (Scheme 56A). A spiro-γ-butyrolactone containing derivative (**252**) was synthesized in seven steps with an overall yield of 15% (Scheme 56B). Beginning with 1,6-dicyclohexene, they obtained the bicyclic compound (**254** and **255**). By utilizing manganese(III) acetate, spirolactonization with 5-*exo*-*dig* cyclization provided spirolactone (**258**) with a yield of 61%. It was noted that this step was crucial for obtaining the final skeleton (**252**). Subsequently, treating (**258**) with SmI_2_ for the reduction of carbonyl to alcohol was followed by acetylation to obtain the final natural compound, (±)-*9*-*acetoxyfukinanolide* (**252**) [121].

## 4. Oxidation Reactions

**Scheme 57:**
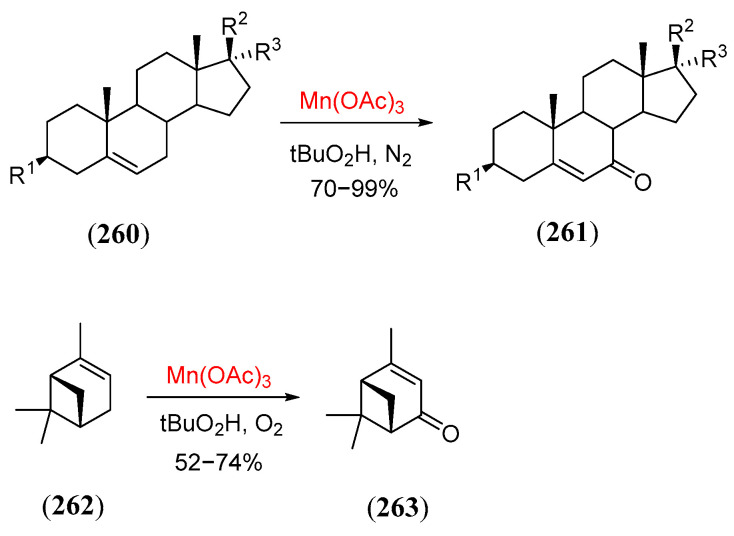
Allylic oxidation of steroids and simple alkenes with manganese(III) acetate.

Manganese(III) acetate can also function as an oxidation agent. Shing reported the synthesis of enones (**261**) using steroids (**260**), where the steroids act as bioactive agents under a nitrogen atmosphere (Scheme 57). Reaction yields were reported to range from 70% to 99%. Conversely, allylic oxidation was employed with simple alkenes (**262**) under an oxygen atmosphere, resulting in the formation of enone compounds (**263**) with good yields ranging from 52% to 74% [122].

**Scheme 58:**
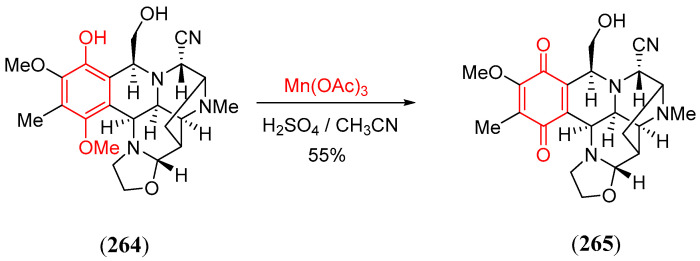
Conversion of phenol to quinone to obtain *cyanocycline A* (**265**).

The oxidation of phenol to quinone may be achieved using manganese(III) acetate in H_2_SO_4_/CH_3_CN at room temperature. Fukuyama reported the synthesis of (±)-*cyanocycline A* (**265**). In the final step of the total synthesis, the phenol group of (**264**) was converted into the quinone (**265**) with a 55% yield by using excess Mn(OAc)_3_ (Scheme 58) [123].

**Scheme 59:**
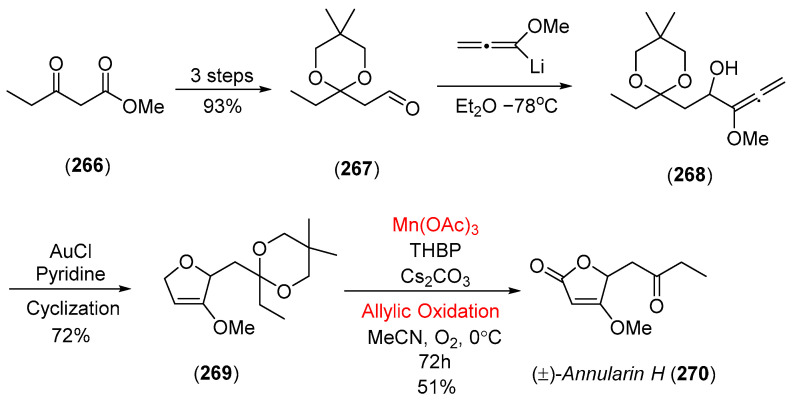
Synthesis procedure of (±)-*annularin H* (**270**).

Brasholz and Reissig proposed the synthesis of (±)-*annularin H* utilizing manganese(III) acetate allylic oxidation to obtain β-alkoxybutenolide derivatives with good yields (Scheme 59). The synthesis procedure started with methyl 3-oxavalerate (**266**), which was transformed into an aldehyde (**267**). Subsequently, it was reacted with lithium alkoxyallene to form an α-allenyl alcohol (**268**), followed by a 5-*endo* cyclization with Au(I) salt, resulting in the synthesis of 2,5-dihydrofuran (**269**). Finally, allylic oxidation afforded the γ-lactone derivative (±)-*annularin H* (**270**). This synthesis enabled the facile production of (±)-*annularin H* in a few steps by employing manganese(III)-based allylic oxidation [124].

## 5. Aromatization

**Scheme 60:**
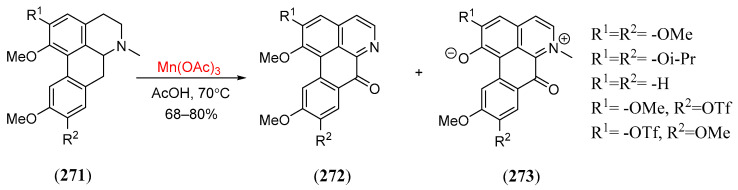
Synthesis of *oxoaporphines* (**272** and **273**) from *aporphines* (**271**).

Singh et al. described the synthesis of *oxoaporphines* (**272** and **273**) from *aporphines* (**271**) using various oxidation reagents, including Pb(OAc)_4_, HIO_4_, PhI(OAc)_2_, and Mn(OAc)_3_-mediated oxidation reactions, resulting in various yields (Scheme 60). However, the best yields were achieved using manganese(III) acetate, ranging from 68% to 80% [125].

## 6. Acetoxidation

**Scheme 61:**
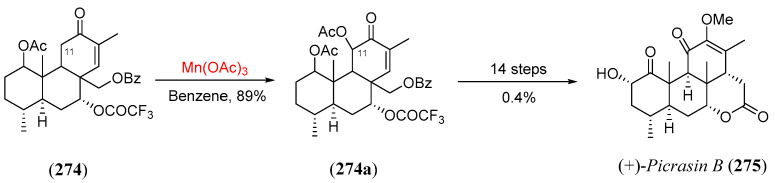
Acetoxidation of C-11 carbon in (+)-*picrasin B* (**275**).

Demir and colleagues initially introduced a one-step method to obtain α’-acetoxidated enones, utilizing manganese(III) acetate or manganese(II) salts of corresponding carboxylic acids [32]. This one-step α’-acetoxidation has been widely employed in various syntheses to selectively substitute alcohol groups in cyclic structures with enone functionality. This method operates via a radical mechanism. Kawada and co-workers utilized manganese(III) acetate to protect and obtain the target molecule at the C-11 carbon (**274**) by transforming it into (**274a**) through acetoxidation in the enantioselective total synthesis of (+)-*picrasin B* (**275**) (Scheme 61) [126].

## 7. Halogen Transfer

**Scheme 62:**
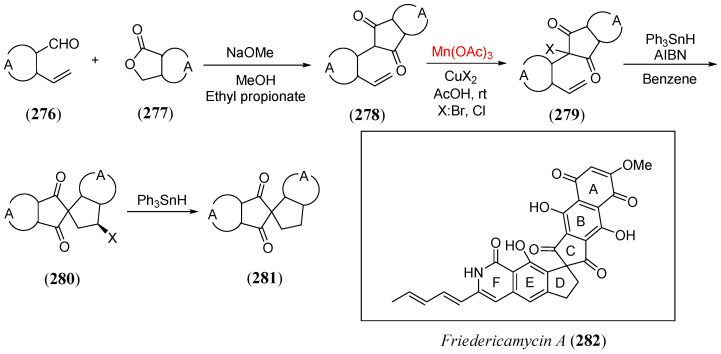
*Fredericamycin A* (**282**) natural product having spiro[4.4]nonane system.

Rao et al. reported the synthesis of *fredericamycin A*, a spiro-natural compound with a spiro[4.4]nonane skeleton (Scheme 62), utilizing free radical cyclization. They employed a radical halogen transfer mechanism within manganese(III) acetate, followed by reductive elimination to construct the spiro[4.4]nonane skeleton (**281**). A range of starting molecules was investigated to understand their effects [127]. The radical halogen transfer mechanism was illustrated in Scheme 62. Halogen transfer facilitated the formation of the spiro-system, yielding only one isomer. According to the authors, this procedure enabled the synthesis of *fredericamycin A* (**282**).

## 8. Polymerization

Hwang et al. conducted the polymerization of a derivative of lignin, *polyguaiacol*, using manganese(III) acetate starting from guaiacol. The reaction was carried out in water and other organic solvents miscible in water, and the effects on polymerization yields were compared [45]. Selective radical polymerization occurred in the synthesis, resulting in a higher yield compared to other polymerization methods. An average molecular weight of 1460 g/mol was reported in the acetonitrile–water solvent solution with an 88% yield, which is significantly higher than those obtained in other organic solvents. In addition to the polymerization of *guaiacol*, other oxidation products were also observed to be formed.

## 9. Conclusions

In conclusion, the synthesis of natural compounds poses significant challenges due to their inherent complexity, often resulting in variations in structure and configuration. Many researchers aim to approximate the final structure in their synthesis attempts, recognizing the difficulty of achieving exact replications consistently. In my assessment, the most challenging aspect of synthesis lies in achieving the appropriate configuration during cyclization. Specifically, manganese(III) acetate not only facilitates cyclization, but also enables stereocontrolled synthesis [113].

Additionally, utilizing manganese(III) acetate proves to be a valuable tool in this regard, as it not only facilitates intramolecular and intermolecular cyclization, but also enables aromatization, polymerization, halogen transfer, acetoxidation, oxidation, and rearrangement reactions, streamlining the process and yielding higher success rates compared to alternative techniques.

Cyclization with manganese(III) acetate typically reduces the number of steps required compared to alternative techniques such as nucleophilic or electrophilic addition. Moreover, manganese(III) acetate cyclization tends to yield higher yields compared to other methods. In summary, radical oxidation reactions based on manganese(III) acetate represent a significant synthetic advancement in organic chemistry. Overall, manganese(III) acetate offers a versatile and efficient approach to forming C–C bonds, indicating a significant advancement in organic chemistry through radical oxidation reactions.

## Data Availability

The data presented in this study are available on request from the corresponding author.
